# Behavioural and cognitive changes in aged pet dogs: No effects of an enriched diet and lifelong training

**DOI:** 10.1371/journal.pone.0238517

**Published:** 2020-09-16

**Authors:** Durga Chapagain, Lisa J. Wallis, Friederike Range, Nadja Affenzeller, Jessica Serra, Zsófia Virányi

**Affiliations:** 1 Clever Dog Lab, Comparative Cognition, Messerli Research Institute, University of Veterinary Medicine Vienna, Medical University of Vienna, University of Vienna, Vienna, Austria; 2 Domestication Lab, Konrad Lorenz Institute of Ethology, University of Veterinary Medicine Vienna, Vienna, Austria; 3 Department of Livestock and One Health, Institute of Infection, Veterinary and Ecological Sciences, University of Liverpool, Liverpool, United Kingdom; 4 Department of Ethology, Eötvös Loránd University, Budapest, Hungary; 5 Department/Clinic for Companion Animals and Horses, University of Veterinary Medicine Vienna, Vienna, Austria; 6 Royal Canin Research Centre, Aimargues, France; University of Illinois, UNITED STATES

## Abstract

Dogs demonstrate behavioural changes and cognitive decline during aging. Compared to laboratory dogs, little is known about aging in pet dogs exposed to different environments and nutrition. In this study, we examined the effects of age, an enriched diet and lifelong training on different behavioural and cognitive measures in 119 pet dogs (>6yrs). Dogs were maintained on either an enriched diet or a control diet for one year. Lifelong training was calculated using a questionnaire where owners filled in their dog’s training experiences to date. Before commencing the diet and after one year of dietary treatment, they were tested in the Modified Vienna Canine Cognitive Battery (MVCCB) consisting of 11 subtests to examine correlated individual differences in a set of tasks measuring general, social and physical cognition and related behaviours. Fourty two behavioural variables were coded and were subjected to principle component analyses for variable reduction. Twelve subtest level components and two Z-transformed variables were subjected to exploratory factor analysis which resulted in six final factors: Problem solving, Trainability, Sociability, Boldness, Activity-independence and Dependency. Problem solving, Sociability, Boldness, and Dependency showed a linear decline with age, suggesting that the MVCCB can be used as a tool to measure behavioural and cognitive decline in aged pet dogs. An enriched diet and lifelong training had no effect on these factors, calling attention to the fact that the real world impact of nutritional and other interventions in possibly counteracting the effects of aging, should be further investigated in pet dogs living under diverse conditions, in order to understand their ultimate effects.

## Introduction

As we grow older, we experience changes in speed processing [1–3], attention [4, 5], memory [6], reasoning [7], executive functioning [8–10], personality [11], emotion [12] and motivation [13]. Similarly, in dogs, aging leads to a decline in learning, memory, attention, executive control, changes in social responsiveness and curiosity towards novel objects [14–21]. Studies examining normal aging in dogs have revealed reductions in their levels of sociability, boldness, playfulness, activity/excitability and trainability [22–25]. Interestingly, aged dogs, like humans, strongly vary in their rate of cognitive decline. In humans, based on this variability, individuals are categorised either as successful agers, displaying mild cognitive impairment or as demented [26]. In a similar fashion, some dogs age in a way that may not directly affect their day-to-day functioning as pets, or their activities and relationship with their owners (successful agers), while another part of the aging dog population develops behavioural changes, cognitive impairment and dementia comparable to neuro-pathological aging in humans (Alzheimer’s disease) [27]. The majority of aging studies have been carried out on laboratory dogs, but recently the focus has been slowly shifting towards pet dogs [28]. Laboratory dogs live in a consistent and controlled environment, whereas pet dogs share the human environment and lifestyle. This includes being exposed to the same pollutants and risk factors of infections as well as a broad range of behavioural and cognitive challenges, and in many cases comparable physical activity levels [28]. Therefore, pet dogs are considered as a more suitable model than laboratory dogs to study different physiological and psychological changes that accompany human aging, and to test various preventive approaches that may hinder cognitive decline.

Although there is a growing body of literature on the cognitive capacities of dogs in general [29], assessment of the behavioural and cognitive changes that accompany aging in pet dogs is scarce. In particular, experimental models suitable to detect age-related behavioural changes and cognitive decline in pet dogs are lacking [16, 30–32]. Some neuropsychological and cognitive tests (such as object, size, picture, landmark, oddity discrimination tasks and Delayed Non-Match to Position task) that have previously been used in humans have been modified and widely used mainly in laboratory dogs; however, few have been applied in pet dogs. Since the tests were developed in a laboratory setting on Beagles, they often require an extensive pre-training protocol and a substantial amount of time to finish training and to complete the task. Therefore, these tests are not feasible to be conducted with pet dogs living in a private household. Alternately, several observational screening questionnaires targeting the behavioural and cognitive changes related to aging have been developed and are commonly used in pet dogs in clinical settings [33, 34]. These questionnaires are filled in by the owners’ or care-takers and are designed to assess their dogs’ behavioural and cognitive changes over time. Based on the scores obtained in different behavioural categories, dogs are classified either as successfully aging, mildly cognitively declining or suffering from cognitive dysfunction (i.e. a syndrome of alteration of various behaviours and cognitive abilities that is different from the norm). Although widely used, individual rating (via questionnaires [33, 34]) relies on the owners’ or care-takers experience of the dogs’ behaviour, which may be associated with biases or different interpretations by different raters [35]. Therefore, it is desirable to develop methods to assess behavioural and cognitive changes by administering a battery of fast and simple tests suitable to determine the profile of behavioural and cognitive deficits in aged dogs. The major advantage of using a test battery is that it can be standardized and so allows objective coding of clearly defined behavioural reactions [35].

Our research group in Vienna has recently developed different tests to measure attention, learning and memory in a sample of 145 pet Border collies aged between 6 months and 14 years [16, 30]. These tests have proven suitable to detect cross-sectional age group changes in attention and learning in Border collies. Importantly, a subsequent study has confirmed that the methods can detect age-related decline in attention across various breeds even if only aged dogs (6 years and above) were tested [14]. Moreover, our group has also developed a test battery to measure personality (consistent behavioural traits) in 217 pet Border collies aged between 5 months and 15 years [35] that we also used to analyse the effects of age on these different behavioural traits (Turcsán et al. submitted). In the current study, we used a modified version of the Vienna Canine Cognitive Battery (VCCB) complemented with tests from the Vienna Dog Personality Test (VIDO-PET) [35] in order to investigate the aging of a wide range of cognitive and behavioural characteristics of dogs that are especially relevant to dog-owner interactions and their relationship. We were interested in measuring any factors which might influence dogs’ performance in independent and social problem solving, learning and trainability. We have termed these factors “basic control processes”, which include individual control processes (measures of motivation, persistency, inhibition, flexibility, and impulsivity), and social control processes (dependency on the owner/humans, and help seeking behaviour). Most of these terms are usually referred to in the literature as being connected to the study of attachment, personality and cognition in dogs.

Furthermore, we also evaluated whether different behavioural and cognitive changes in aged pet dogs are affected by a nutritional intervention and lifelong training. Nutritional supplements and lifelong training are thought to delay cognitive aging in humans and dogs [36]. Interventions that utilize extrinsic factors such as environmental enrichment including social, physical and behavioural/cognitive stimulation as well as dietary/nutritional interventions may protect against age-related neuropathology and cognitive decline in dogs [36]. Physical exercise and cognitive training induce both temporary and permanent changes at the structural and functional levels in the aging brain [37–42], which has been supported by neuroscientific evidence [43]. Combining physical activity and cognitive training in interventions produces major benefits to older humans’ cognitive and physical functions, health status, emotional status and wellbeing [44]. In line with these human results, lifelong training helped to retain measures of attentiveness in senior dogs [14]. In laboratory Beagles, a combined treatment of behavioural enrichment (consisting of exercise and cognitive training) and an antioxidant diet has proven to be more effective in attenuating age-dependent cognitive decline than cognitive training alone [36, 45]. Reports of the effects of lifelong training on a broader cognitive profile in pet dogs are currently lacking.

Since aging is associated with a progressive accumulation of oxidative damage in the brain, the use of antioxidants such as Vitamin E, Vitamin C, beta-carotene, flavonoids and polyphenols can reduce the level of oxidative damage and delay age-related cognitive decline [46]. Nutritional antioxidants act as free radical scavengers by directly neutralizing free radicals, or reducing the peroxide concentrations and repairing oxidized membranes [47]. Several cross-sectional and longitudinal studies in laboratory beagles have reported the positive effects of antioxidant fortified food in attenuating cognitive decline measured in different domains like attention, learning, cognitive flexibility and executive functions [36, 45, 48–52] but see Snigdha et al. [53] for negative results. Moreover, reduced oxidative damage and Aβ plaque pathology in the brain, as well as reduced mitochondrial dysfunction have been reported in laboratory dogs fed with an enriched diet containing antioxidants and mitochondrial enzymatic co-factors [46, 54, 55]. Contradictory results about the effectiveness of antioxidant supplementation have been documented in humans. Some studies have reported improved memory function [56, 57], while others have failed to show improvement in cognition [58, 59].

Another ingredient, omega-3 fatty acid (Docosahexaenoic acid (DHA)) frequently used as a dietary supplement, is thought to enhance cognitive abilities by facilitating synaptic plasticity and/or enhancing synaptic membrane fluidity. Moreover, it might also act through its effects on metabolism, as DHA stimulates glucose utilization and mitochondrial function by reducing oxidative stress (see review by [60]). In humans, increased fatty fish and n-3 Long Chain Polyunsaturated fatty acids (LCP) consumption is associated with a reduced risk of impaired cognitive function [61]. Similarly, Phosphatidylserine acts as a neuro-protector against degenerative processes during aging. Osella et al. [62] reported the beneficial effects of Phosphatidylserine in improving cognitive functions, such as memory, orientation, learning, and social behaviour in pet dogs. Finally, tryptophan which is a common ingredient in dietary supplements, has been proposed to reduce aggression in dogs [63]. Old animals may become more aggressive due to impaired senses, physical debilitation or painful conditions [64], so the addition of tryptophan to the diet may help in the behavioural modification of old dogs. In the current study, the animals were fed with a diet enriched in antioxidants (Vitamin C, Vitamin E & Polyphenols), DHA, Phosphatidylserine and tryptophan, and we examined whether this nutrient cocktail had any effect on the behavioural and cognitive measures of pet dogs aged over 6 years. Since this was a nutrient cocktail, we could not disentangle the effects of single ingredients; hence, we proposed that the combination of the ingredients in this nutrient cocktail would help to slow down the aging of cognitive and behavioural functions. The objectives of the present study were: 1) to evaluate cross-sectional behavioural and cognitive changes that appear with age using a newly created battery of tests and 2) to assess the effectiveness of an enriched diet in counteracting age-dependent behavioural and cognitive changes in comparison to a control diet (both diets consisted of an age-appropriate maintenance food, only the test diet was fortified with a broad spectrum of antioxidants, DHA, Phosphatidylserine and tryptophan), 3) to evaluate whether lifelong training helps to slow down the aging of different behavioural and cognitive measures in aged pet dogs. By assessing the different behaviours and cognitive abilities of a cohort of dogs in the present study using the Modified Vienna Canine Cognitive Battery (MVCCB), we hope to provide a useful and practical tool to detect behavioural and cognitive changes in aged pet dogs, which may help to diagnose and treat affected individuals early in the disease process.

## Material and methods

### Ethical statement

The institutional ethics and animal welfare committee at the University of Veterinary Medicine Vienna (Protocol number: 05/03/97/2014) approved this experiment. All dog owners signed a consent form at the start of the study.

### Animals

One hundred and nineteen pet dogs from a total of 30 different breeds and mixed breeds were recruited for the study. The recruitment of dogs was conducted through word of mouth, distributing flyers and screening dogs over 6 years from the Clever Dog Lab (CDL) database. The average age of the dogs at the start of the study was 9.1 years (range: 6.1–14 years (73–168 months)), and the average weight was 22 kg (range: 7–42 kg). Age-related cognitive deficits are known to start as early as 6 years of age in beagle dogs [65], therefore we recruited dogs older than 6 years.

Before enrolment in the study, all dogs attended initial veterinary examinations including physical, orthopaedic, neurological and ophthalmologic examinations. Additionally, a complete blood count and blood chemistry profile was performed on each dog to ensure that they were healthy and eligible to participate. Dogs with changes in mobility suspected to be due to osteoarthritis or other underlying painful conditions and/or moderate to severe impairment of visual or auditory capacity were not included. Owners also filled in a Canine Cognitive Dysfunction Questionnaire (CCDR, Salvin et al. [33]) at the beginning of the study and after a one year period. The questionnaire consisted of questions that refer to disorientation, decreased social interactions with owners, other pets and the environment, sleep-wake cycle disturbances, house soiling, anxiety problems and changes in activity levels. All the questions were scored based on severity (as described by Salvin et al. [33]), and a total dysfunction score (Canine Cognitive Dysfunction (CCD)) was obtained by summing all scores. We used this questionnaire to detect dogs suffering from Canine Cognitive Dysfunction at the beginning and to monitor any changes in dogs’ behaviour after the one year period. However, based on their final score, none of the dogs were categorized as having CCD. Recruited dogs also received an additional veterinary examination and blood test after one year of dietary treatment (see below). A detailed description of the subjects, including dogs that dropped out during the study period is presented in the S1 and S2 Tables in S1 File.

After enrolment in the study, owners filled in an extensive demographic questionnaire that included details of their dog’s lifelong training experiences in 13 different types of training (puppy school, obedience, agility, BGH (Begleithund), protection dog training (Schutzhund), service dog training, search and rescue training, dog dancing/trick training, dummy training, hunting/nose work, sheep dog training, therapy dog training, and other). For each training type, the owners were asked to give a score based on their past and current training attendance as follows: no experience = 0, sporadic training = 1, once or twice a month = 2, once or twice a week = 3 and completed training (with or without an exam) = 4. A lifelong training score was then calculated for each dog by summing up all the 13 scores and could range from 0 to 52; dogs with “0” score having no formal training and “52” with the highest training. The average lifelong training score of dogs was 11.55 (range = 0–34).

### Treatment

The study was conducted as a randomised, double-blinded, controlled study. Dogs were divided into two groups (N1 = 60 & N2 = 59) matched for age, sex, lifelong training score, weight and breed (S1 and S2 Tables in S1 File). Each group received one of two diets i.e. “diet 1 (round kibble (test diet))” or “diet 2 (square kibble (control diet))” for a period of one year. Even if the majority of studies conducted with laboratory beagles and pet dogs have used a feeding duration of less than one year to determine the effectiveness of an enriched diet on behaviour and cognition [36, 49–51, 66, 67], we decided to maintain the dogs on the diets for one year. Before commencing the test and control diets, all the dogs participated in two weeks of a run-in period where they were fed a new diet (“triangle kibble”) to determine their acceptance of the diet. The composition of this diet was similar to the control diet.

The main ingredients of the diets included rice, poultry and poultry by products, wheat, corn, poultry fat, corn gluten, liver hydrolysate, beet pulp, minerals and vitamins, wheat gluten and psyllium. Although the test and control diets were identical in the composition of main ingredients, in the test diet, a small fraction of rice was replaced with antioxidants (Vitamin C, Vitamin E & Polyphenols), omega 3 fatty acids (Docosahexaenoic acid), phospholipids (Phosphatidylserine) and a higher amount of tryptophan. The composition of the test and control diets are presented in Table 1. The caloric density of the test diet was 3826 kcal/kg and the control diet was 3884 kcal/kg. Caloric density was calculated according to the method published in National Research Council [68]. The guaranteed analysis was similar between the two diets and is provided in Table 2. The diets were manufactured by a private pet food company and supplied to the CDL, Vienna. The nutritive intake of each dog was calculated separately based on age, weight and body condition score. Dog owners were provided with food bags every month and were instructed to strictly give the test or control diets as 90% of the dog’s food supply and limit training treats and other food to a maximum of 10%. No feeding of additional dietary supplements was permitted besides the test or control diets. Owners were verbally asked every month during the study about whether or not they were following the feeding guidelines we had provided, and additionally filled in an owner compliance questionnaire at 6 months and 12 months to monitor feeding compliance. The food bags received from the dog food company were labelled either”round” or”square”, and neither the experimenter nor the owners knew the composition until all analyses had been completed.

**Table 1:** Composition of test and control diets.

	Test (enriched diet)	Control
**Moisture (%)**	9.5	9.5
**Crude protein (%)**	25.1	25.3
**Crude fat (%)**	13.4	14.0
**Crude fibre (%)**	1.6	1.7
**Ash (%)**	5.1	4.3
**Tryptophane (%)**	0.45	0.24
TRP/LNAA* **ratio**	0.067	0.036
**DHA (%)**	0.17	0
**Phosphatidylserine (ppm)**	328	0
**Vitamin E (ppm)**	839	499
**Vitamin C (ppm)**	559	0
**Green tea polyphenols (ppm)**	425	0
**ME NRC 2006 (kcal/kg)**	3826	3884

* Large Neutral Amino acids (Tryptophan, Tyrosine, Valine, Leucine, Isoleucine, Phenylalanine).

**Table 2:** Guaranteed analysis of the test and control diets.

Moisture (%)	9
Protein (%)	24.5
Fat (%)	14.1
Ash (Minerals) %	4.3
Crude fiber (%)	1.7
Nitrogen free extract (estimation of digestible carbohydrate) %	45.5

### Test setting and procedure

#### MVCCB 1 (before diet)

Each dog in the study participated in a modified version of the “Vienna Canine Cognitive Battery” (MVCCB) that included 11 subtests measuring general, physical and social cognition, and human-animal interactions. Five of these subtests were taken from the VIDO-PET (exploration, picture viewing, food choice, separation, and the greeting and playing task) [35]. Four subtests were used from the original VCCB (attention, novel action, manipulative persistency, and clicker training for eye contact (Wallis et al. [30], Wallis et al. unpublished study). One of the remaining two subtests was adapted from the selective attention test used by Mongillo et al. [69], and the final subtest was a modified version of the object permanence task with a delay by Gagnon and Doré [70]. The first test battery (MVCCB 1) was carried out to check whether the two treatment groups (diet 1 and diet 2) were comparable prior to starting the diet feeding, as would be expected due to the balanced subject design. The subtests were split into two test sessions (containing 7 and 4 subtests respectively) with 7 to 30 days between sessions. Each session lasted around one hour with breaks between subtests as necessary. The order of the 11 subtests was the same for all dogs. Details of the procedure of MVCCB 1 are provided in the S1 File.

#### MVCCB 2 (after diet)

After one year of dietary treatment, the MVCCB 2 was performed in order to assess the effects of age, lifelong training and diet on different measures of behaviour and cognition. The second battery also included 11 subtests, 10 of which were the same as in MVCCB 1; only the selective attention test from MVCCB 1 was replaced with a detour task in MVCCB 2, which was taken from the original VCCB. All apparatus and set ups in the MVCCB 2 were visually and/or texturally different than in the first test battery to minimize test-retest effects. All the subtests were conducted in a different room (adjacent to the room used in MVCCB 1). The rooms had the same size (7.12m x 6m), and the same layout. The same experimenter (hereafter “E”; main author DC) carried out MVCCB 2 as well as MVCCB 1 with the exception of the greeting and playing task where other female “strangers” were used compared to MVCCB 1, because after one year, the E was considered to no longer be a stranger to the dogs. In the testing room, two doors were located approximately two meters apart on the front wall. One door was designated as the owner door, and she/he used this door exclusively to enter and exit the room, the other door was the experimenter’s door. Each door location was marked with a 1 meter semi-circular taped section on the floor, to aid in determining the dogs’ position relative to the doors. The room contained only the equipment required for the actual test. Owners were instructed verbally to follow the experimenter’s instructions for each subtest and were additionally handed a written test protocol, which they read before each subtest. The subtests are described briefly below with a short description of all variables that were measured. For a full list of variables measured in each subtest, please refer to Table 3.

**Table 3:** Detailed description of all behavioural variables measured in 11 subtests in MVCCB 2.

Name of subtests	Variables coded	Type of variable	Description	Inter-observer reliability (Cronbach’s alpha/ Cohen’s kappa)
**Exploration**	Exploration	Percentage time	Dog was moving/standing in the room and had its nose on or in close proximity (approx. 5 cm) to the floor/wall/object/s or both front paws placed on an elevated surface (e.g., window sill, table).	0.95
	Locomotion	Percentage time	Dog was moving in the room with or without any directed activity.	0.99
	Inactive	Percentage time	Dog was inactive (sitting/standing/lying).	0.99
	Position at O_1m	Percentage time	Dog was standing/sitting/lying or moving within 1m of O.	0.94
	Looking at O	Percentage time	Dog’s head and eyes were orientated towards O (dog was moving or stationary).	0.97
**Picture viewing**	Follow O	Duration	Dog was moving in the direction of the moving O or stationary O.	0.95
	Move independent	Duration	Dog moved independently of O.	0.90
	Position at O_1m	Duration	Dog was standing/sitting/lying or moving within 1m of O.	0.96
	Looking at O	Duration	Dog’s head and eyes were orientated towards O (dog was moving or stationary).	0.94
**Food choice**	No. of correct choices	Frequency	Number of times the dog chose the plate with sausage in both step1 and step2.	100
**Separation**	Position at door	Duration	Dog was positioned within the semi-circle in front of one of the two doors.	0.96
	Look at door	Duration	Dog was sitting/standing/lying outside the semi-circle but looking at one of the two doors.	0.98
	Locomotion	Duration	Dog was moving in the room with or without any directed activity.	0.98
	Exploration	Duration	Dog was moving/standing in the room and had its nose on or in close proximity (approx. 5 cm) to the floor/wall/object/s or both front paws placed on an elevated surface (e.g., window sill, table).	0.97
**Greeting and Playing**	Latency to approach S/O	Latency	Measured from the moment the dog approached S/O within 20cm after S/O entered the room and stood by the door.	0.99
			This variable was coded separately for S and O.	
	Greeting (S/O)	4-point scale	0: dog did not approach/approached initially but then avoided (with no interaction with S/O). 1: slowly sniffed or passively stood after approaching S/O with or without tail wagging. 2: friendly greeting, tail wagging, may have cuddled up, jumped or licked. 3: very excited/enthusiastically greeted with intensive searching for contact and tail wagging.	0.98 Cohen’s kappa
			This variable was coded separately for S and O.	
	Playing (S/O)	4-point scale	0: no play. 1: mouthed toy sometimes but did not play/ may have played after a while but needed some encouragement. 2: mouthed toy and sometimes pulled it/brought it back. 3: played more than 90% of time.	0.99 Cohen’s kappa
**Memory test with distraction**	Latency to find food	Latency	Measured from the first detectable forward movement of the dog (after O removed the lead) until the dog found the food.	0.99
**Detour**	Latency to success	Latency	Latency to get the reward in trial 1, 3 and 4. This variable was coded separately for each trial.	100
	Time close to gate	Duration	Duration of time dog stayed close to the gate (the head of the dog was within 50 cm of the gate) in trial 1, 3 and 4. This variable was coded separately for each trial.	0.98
	Looking at E	Duration	Duration of time dog looked at E in trial 1, 3 and 4. This variable was coded separately for each trial.	0.98
	Looking at O	Duration	Duration of time dog looked at O in trial 1, 3 and 4. This variable was coded separately for each trial.	0.98
**Attention**	Time looking at toy/human (E)	Duration	Total duration of time the dog looked at the moving toy/human (E). This variable was coded separately for the toy and human condition.	0.98/0.98
	Time looking at O	Duration	Total duration of time dog looked at O. This variable was coded separately for the toy and human condition.	0.97/0.98
**Novel action**	Latency to pull out board	Latency	Measured from the point when the yellow board was visible until the dog pulled out the board and ate the sausage in trial 1.	100
**Manipulative persistency**	Manipulate toy	Percentage time	The toy was pushed or moved with the dogs’ nose or paw (only actual contact with the toy was measured) in step 1 and step 2 separately.	0.95
**Clicker training for eye contact**	Latency to eye contact	Latency	Measured from the moment the dog had taken the sausage into its mouth until the dog looked up into the face of E. The average of the first 3 trials and average of the last 3 trials were measured as separate variables.	0.96
	Latency to find food	Latency	Measured from the moment the piece of sausage left E’s hand, until the dog found the food, and took it into its mouth. The average of the first 3 trials and the last 3 trials were used as separate variables.	0.72

#### Exploration

Aim: to assess the dogs’ general activity and exploration in an unfamiliar environment.

Procedure: The room was equipped with a few objects like a plastic dog crate, a bag of folded newspapers, a small open suitcase containing rugs, a toy watering can, bean bag, a plastic kite and a table with a closed box of sausage placed on the top. The Owner (hereafter “O”) entered the room with his/her dog on a lead, took off the lead at the centre of the room and indicated that the dog was free to explore the room while he/she remained standing in the same place for 2 minutes. O did not pay any special attention to the dog and did not interact with the dog. After 2 minutes, E signalled a beep tone from outside the room to indicate that the test was over. We coded/investigated variables measuring locomotion, exploration, staying within 1 meter of O, looking at O, and time spent inactive as measures of the dogs’ spontaneous reaction to a new environment.

#### Picture viewing

Aim: to assess both activity and movement relative to the owners’ movement.

Procedure: After hearing the beep sound, O walked towards the first dog picture located on one of the walls, paused to look at it for 5 seconds, and then continued to walk around the room, looking at all the seven pictures on the walls in turn, while ensuring that he/she spent 5–6 seconds in front of each picture. While walking, he/she ignored the dog, no eye contact was made. After 1 minute, E signalled a beep tone to indicate O to go to the centre of the room and wait for the next test. In this test, we measured if the dog moved independently or followed O, looked at O, and stayed within 1 meter of O.

#### Food choice

Aim: to assess food motivation and dogs’ dependence on the owner by analysing how much the owner’s choice influences the dog’s choice.

Procedure: Following the picture viewing test, E entered the room and started the task. O sat on a chair on one side of the room reading a newspaper and the dog was leaded to a hook on the wall on the other side. After pre-training, in which dogs had the opportunity to obtain a piece of sausage from a single plate on the floor, which was kept 1.5 meter from the dog, the food choice test was conducted in two steps. In the first step, over 6 consecutive trials, E showed the dog two yellowish green coloured plates, one with two pieces of sausage and the other plate being empty. E placed both plates on the floor an arms width apart, 1.5 meter from the dog, released the dog and recorded the first visited plate choice. In the second step, O was asked to show a clear preference for the empty plate over the other plate with sausage on it, in order to see whether the dog’s choice will be influenced by its O. This was done over 6 consecutive trials; after E placed the plates on the floor, O got up from his/her chair, crouched close to the empty plate, picked it up and acted as if it was really interesting and delicious for the dog, saying “yum yum yum”. Then O placed the plate back on the floor and returned to his/her chair. Afterwards E released the dog again and recorded the dog’s choice. The location of the sausage was counterbalanced between trials and the same location was never baited for more than two consecutive trials. A more detailed description of this test can be found in Turcsán et al. [35]. In each of the two steps we counted the trials (out of 6) in which the dog chose the plate with the 2 pieces of sausage on it.

#### Separation

Aim: to assess the dogs’ reaction to separation and activity when left alone in the room.

Procedure: Following the food choice task, both O and E left the room using their respective doors and closed both doors while the dog stayed behind for 2 minutes. O did not give any command to the dog when leaving. We measured the duration of time the dog stayed close to each door or looked at the doors, and the time spent being active by measuring locomotion and exploration.

#### Greeting and playing

Aim: to assess the dogs’ attraction to and playfulness with a stranger as compared to the owner.

Procedure: After 2 minutes of separation, the stranger (hereafter "S"), who was unfamiliar to the dog entered the room, closed the door and stood close to the door without interacting with the dog for 5 seconds, after which she crouched down, called and greeted the dog either by petting when approached or by talking in a friendly manner if the dog remained distant. S then walked towards the window, took a rope toy from the windowsill and attempted to play a friendly tug-of-war game with the dog for 30 seconds. After 30 seconds, E knocked on the door signalling S to leave the room. After 30 seconds, O entered, greeted and played with the dog using the same protocol as S. We scored the greeting and playing behaviour of the dog towards S and O separately.

#### Memory test with distraction

Aim: to measure the dogs’ persistence to search for a hidden food reward as well as their ability to remember its location following a three minute delay during which the owner intentionally distracted the dog (based on Gagnon and Doré [70]).

Procedure: From the starting position, E showed the dog (held on a lead by O) two pieces of sausage and then walked backwards continually calling the dogs’ name and hid the pieces behind one of two wooden panels placed in the corner of the testing room at a distance of 4.5 meters from the dog. After hiding the sausage, E then looked at the wooden panel and performed a proximal pointing towards it for 5 seconds. O then went outside with the dog and waited for 3 minutes while positively trying to engage the dog by doing some tricks and commands to cause some distraction while E stayed in the testing room. Thereafter, O returned with the dog and released it from the starting position to find the hidden food for a maximum of 2 minutes and then again left the room. We measured the latency to find the food in this task.

#### Detour

Aim: to measure dogs’ ability to solve a problem individually or with human social support.

Procedure: The apparatus consisted of two parallel fences that were positioned at the back of the testing room and were open at one end. A gate was located along the right wall, in front of the owner’s chair. The gate could be open or closed depending on the trial. E always placed the reward (two pieces of sausage on a plate) between the two fences at the closed end, behind the gate, before the dog and the owner entered the room in all trials. Dogs were tested in four trials regardless of whether they succeeded or failed to solve the detour. In the first trial, O entered the room with the dog and sat on a chair positioned 1.5 meters from the closed gate with the dog held next to him/her. E then signalled O to release the dog. O could encourage the dog but was not allowed to point with his/her hands/head or give verbal instructions directing the dog. If successful in solving the task within 2 minutes, the dog was allowed to eat the sausage, if not, O put the dog back on the lead and was instructed to leave the room. Trials 1 and 3 were identical set ups, whereas in trial 2 the gate was open. In trial 4, the gate was closed but this time E demonstrated the detour while continuously calling the dog’s attention and showing the dog two large pieces of sausage in her hands. Once she reached the plate, she placed the sausage on it, and walked back to her original position holding out her hands to show that she no longer had the treat. After she returned, the dog was released to find the reward. If the dog managed to get around the fence to the reward, it could eat it. If the dog failed to solve the detour in 2 minutes, E indicated that O should call it back to him/her. E then showed the dog the correct way to make the detour, so that the dog got the reward in this task before she left the room with the dog. We measured different variables such as the latency to reach the food, total duration of time spent in front of the gate and looking at E and O in trials 1, 3 and 4.

#### Attention

Aim: to measure the dogs’ sustained attention towards two different stimuli (a moving toy train and human).

Procedure: O entered the room with the dog on a lead, tied the dog to the red lead attached to a holder on the floor, sat down on a chair and pretended to read a test protocol. Two conditions were presented in front of the dog (at a distance of 4.5 meters) in a counterbalanced order: a moving toy train and a human moving in circles. In the toy train condition, O was instructed to press the start button of the toy´s remote control when the dog was looking away from the toy train. The battery powered toy train followed the oval track moving in one direction. After 2 minutes when E signalled from outside by knocking on the door, O pressed the stop button of the remote control and left the room with the dog. In the human condition, after O and dog were in position, E entered the room and walked through the room without paying attention to the dog. When she reached the back wall, E started to walk slowly in circles in a marked area (similar to the location of the train) for 2 minutes. After 2 minutes, E left the room and signalled O to leave the room with the dog. We measured the total duration of looking at the toy and human, and looking at O in both conditions.

#### Novel action

Aim: to measure dogs’ ability to solve the task by pulling out a board and eating the reward.

Procedure: The apparatus used for this experiment consisted of a black wooden board (80 x 65 cm) with two metal rails positioned in lowered depressions. A yellow coloured wooden board (60 x 10 cm) could be mounted onto either metal rail and moved smoothly backwards and forwards, aided by small wheels positioned underneath it. The apparatus was positioned in one corner of the experimental room and surrounded by three fences, which were covered by blankets, creating an enclosure that the dog was not able to enter. The front fence had a 5 cm gap at the bottom where the apparatus could be pushed out enabling access to it when manipulated by the dog. E was positioned inside the fenced enclosure. The yellow board was positioned either on the left or the right side of the apparatus, and the order was counterbalanced among dogs. E pushed out the yellow board, so that just the first 10 cm was visible to the dog. She then placed food (sausage) on the board out of sight of the dog or O; however, the dog was able to smell the food. In the first trial, the food was placed close (10 cm) to the visible end of the yellow board, and the dog had to use its paw to obtain the reward by pulling out the board. Verbal encouragement was allowed by O. After successfully pulling out the board and eating the reward, E swapped the board to the other side of the apparatus, and the dog was asked to solve the same task again. From the second trial, the sausage was placed 31 cm from the end of the yellow board that was visible to the dog. In total, 11 trials were carried out if the dog successfully pulled out the board in each trial. If the dog was unsuccessful after trying for five minutes, then the trial was ended, and no further trials were presented. We measured the latency to pull the board in trial one and 10 subsequent trials; however, we only used the measurement of the first trial in the final analysis due to the variation of total number of completed trials.

#### Manipulative persistency

Aim: to measure dogs’ motivation and persistence in a solvable and unsolvable task.

Procedure: In this task, the dog was provided with a manipulative toy called “Twist and Treat” (PetSafe Busy Buddy) filled with food. The dog could play with it for 4 minutes divided into two 2-minute steps. In step 1, the toy was filled with small pieces of cheese so that when the dog manipulated the toy, the cheese came out. If the dog lost interest in the toy, O could encourage it verbally from his/her chair. After 2 minutes, E picked the toy up and filled it with bigger pieces of cheese that did not fall out during toy manipulation. E gave the toy back to the dog that could again have 2 minutes to manipulate the toy (step 2). As in step 1, if the dog lost interest in the toy, O could encourage it verbally. We measured the duration of time dogs spent manipulating the toy in step 1 and step 2.

#### Clicker training for eye contact

Aim: to measure dogs’ selective attention towards the human and reward, and trainability of dogs by comparing performance in the last three trials and the first three trials respectively.

Procedure: In this task, dogs had to perform two tasks consecutively and switch between two responses. At first, they were required to find a small piece of sausage dropped on the floor by E, then after eating the food, E waited for the dog to establish eye contact with her. These two tasks were repeated over a period of five minutes. To aid the dogs in learning the task, when they established eye contact, E marked the behaviour using a clicking sound produced by a "clicker". A detailed description of this test can be found in Chapagain et al. [14] and Wallis et al. [30]. We measured the average latency to make eye contact with E and the average latency to find food on the floor in the first three trials and the last three trials during the five-minute period.

### Data collection

All tests were videotaped using a set-up of four digital video cameras placed inside the room and connected to a video station outside of the testing room. The videos generated from the tests were later coded using the video-coding software Solomon Coder beta 15.11.19 (by Andras Peter; http://solomoncoder.com).

In total, 42 behavioural variables were coded across the 11 subtests of the MVCCB 2 and were used for subsequent statistical analysis: N = 25 durations (N = 7 calculated as percentage time), N = 11 latencies, N = 4 nominal scores, N = 2 other scores (number of correct choices). A description of all coded behavioural variables is presented in Table 3. The analysis done to test the effectiveness of diet on these 42 variables is presented separately in S4 Table in S1 File. A randomly chosen sub-sample of 20 dogs were coded by a second coder, and inter-observer reliability was calculated by calculating Cronbach’s alpha for all variables except “Greeting S/O and Playing S/O”, where we calculated Cohen’s kappa. Inter observer reliability was classed as very good for all the variables measured in the tests (see Table 3).

### Data reduction and generation of PCA components and factor scores from EFA

The variables coded in the MVCCB 2 were subjected to a two-step data reduction procedure according to the methods described by Turcsán et al. [35] in order to allow the exploration of the underlying structure of the behaviours across the subtests. In the first step, the raw behavioural variables in 9 of the 11 subtests were subjected to a PCA with Varimax rotation (in IBM SPSS vs. 24) in order to reduce the number of variables in each subtest, while maximizing the variance retained. In total, 9 PCAs were run which resulted in 13 components where the Eigenvalues were larger than 1. We did not apply a data reduction method in two subtests (memory test with distraction, the novel action test) since only one variable was measured in each of these tests. Therefore, for these two subtests the variables measured were Z-transformed to allow comparison on the same scale.

In the second step, an exploratory factor analysis (EFA) with Varimax rotation (in IBM SPSS vs. 24) was carried out on the 13 PCA components and the two Z-transformed variables extracted from the subtests in the first step of the data reduction. We carried out the EFA to further reduce the number of components, and to examine the correlations across the subtest components. Cronbach’s alpha was calculated to assess the internal consistency of the extracted factors.

Furthermore, we used the PCA and EFA results from the analysis in SPSS as a template to calculate the factor scores for each individual dog, so that we were able to maximise the sample size in each factor by allowing missing values. We used a similar procedure to Turcsán et al. [35], by firstly standardising the raw variables using a Z-transformation, and then calculating the subtest component scores by taking the mean of the variables loading with at least 0.5 on a given component. If a variable loaded negatively on a component, it was first multiplied by -1. For the components that were comprised of three variables, a single missing value per individual was allowed. In the final step, we calculated the EFA scores by taking the means of the subtest-level components loading with at least 0.32 on a given factor. Again, components that loaded negatively were multiplied by -1. For the factors that were comprised of three or more components a single missing value per individual was allowed.

### Statistical analyses

The EFA factors were analysed using general linear models with diet (test and control), age in months, lifelong training score, and the interaction between diet and age in months and diet and lifelong training score included as fixed effects. Analysis of before diet performance of dogs in MVCCB 1 was only done if we found a significant effect of diet in MVCCB 2. Model residuals of the six EFA factors were tested for normality using the Shapiro-Wilk test and residual distribution charts, and homoscedasticity was assessed via plots of residuals against fitted values. The EFA factors were transformed when necessary to fulfil the assumptions of normality and homogeneity of variance. Non-significant predictors (p >0.05) (including trends for interaction effects (p values > 0.05 but less than 0.10) were removed from the model and the models were re-run until we obtained the final model. Before running the model analyses, we checked for a correlation between age in months and lifelong training score and found no correlation (Spearman correlation: r = -0.03, p = 0.78). Therefore, both age in months and lifelong training score were used as covariates in each model. All statistical analyses were performed in R 3.2.2 [71] and the graphical illustrations were created in IBM SPSS statistics V24. In order to determine effect size [partial eta squared (η^2^)] of predictors used in the model, we used R package “sjstats” [72]. According to Richardson [72], Cohen (1969, pp.278-280) provides partial eta squared values of 0.0099, 0.0588, and 0.1379 as benchmarks for small, medium, and large effect sizes respectively.

## Results

Out of the 119 dogs that participated in the study, 99 dogs completed the study. From the remaining 20 dogs, six dogs died during the study period, seven dogs were dropped from the study due to diet related issues, and seven dogs dropped out due to reasons other than diet. Furthermore, the data of another five dogs had to be discarded because the owners did not comply with our feeding guidelines and provided dogs with extra supplements. Therefore, the final analyses included only 94 dogs (45 test diet and 49 control diet, see S1 Table in S1 File). The average age, weight and lifelong training score of the dogs included in the test and control diet groups is presented in the S3 Table in S1 File.

### PCA components generated from the tests included in MVCCB 2

#### Exploration

A PCA carried out on the measured variables for this subtest revealed that they grouped on one component explaining 58.26% of the variance. We labelled this component ‘*Activity*/*Exploration*’, as the variables that strongly loaded on it all measured dogs’ activity levels (Table 4).

**Table 4:** Component derived from PCA on the variables measured in the exploration subtest.

Variable	Activity/Exploration
Percentage time of being inactive	-0.960
Percentage time of locomotion	0.909
Percentage time of exploration	0.780
Percentage time of looking at O	-0.533
Percentage time of within 1m of O	-0.523
Eigenvalue	2.913
Explained variance (%)	58.260

Kaiser-Meyer-Olkin Criterion *(KMO)* = 0.641; Bartlett: χ^2^ = 317.13, df = 10, p < 0.001.

#### Picture viewing

The PCA revealed that the variables measuring duration of time that the dog remained within one meter of O and following O loaded positively in one component, whereas moving independently loaded positively on a second component. Looking at O loaded negatively on both components (Table 5). Looking at O loaded relatively highly on both components. We labelled the first component “*Dependency*” since all the variables were related to O and the second component as “*Independence*”, since moving independently loaded higher in this component. These two components explained 89.52% of the observed variation in the data.

**Table 5:** Components derived from PCA on the variables measured in the picture viewing subtest.

Variable	Dependency	Independence
Duration of time within 1m of O	0.938	
Duration of following O	0.882	
Duration of looking at O	-0.716	-0.603
Duration of moving independently		0.978
Eigenvalues	2.40	1.179
Explained variance (%)	54.25	35.27

*KMO* = 0.546; Bartlett: χ^2^ = 194.48, df = 6, p < 0.001.

#### Food choice

A PCA on the measured variables for this subtest revealed that they grouped on one component explaining a variance of 71.55%. We labelled this component “*Food motivation*”, as the variables measured the number of the dogs’ choices of the baited plate (Table 6).

**Table 6:** Component derived from PCA on the variables measured in the food choice subtest.

Variables	Food motivation
Number of choices of baited plate in step1	0.846
Number of choices of baited plate in step2	0.846
Eigenvalue	1.43
Explained variance (%)	71.55

*KMO* = 0.500; Bartlett: χ^2^ = 18.81, df = 1, p < 0.001.

### Separation

A PCA on the two variables grouped them together on one factor explaining 92.43% of the variance. We labelled this component “*Resisting separation”*, as the variables most likely measured the motivation to leave the room (Table 7). Locomotion and exploration had to be excluded from the PCA since these variables had low commonalities (less than 0.3), which resulted in a KMO of below 0.5. Therefore, the final PCA included only the two remaining variables.

**Table 7:** Component derived from PCA on the variables measured in the separation subtest.

Variables	Resisting separation
Duration of looking at door	-0.961
Duration of positioned at door	0.961
Eigenvalue	1.84
Explained variance (%)	92.437

*KMO* = 0.500; Bartlett: χ^2^ = 116.53, df = 1, p < 0.001.

#### Greeting and playing

The PCA revealed that the two variables measuring playing with S and playing with O grouped on one component, whereas the latency to approach O and greet O grouped on a second, and the latency to approach S and greet S grouped on a third component (Table 8). The first component was labelled as *“Playfulness”* since both variables measured how playful the dogs were with S and O. We labelled the second component as “*Dependency*” since both components were related to O, and the third component “*Openness*” since both variables were related to interacting with S. These three components explained 69.56% of the observed variation in the data.

**Table 8:** Components derived from PCA on the variables measured in the greeting and playing subtest.

Variable	Playfulness	Dependency	Openness
Playing with O	0.887		
Playing with S	0.808		
Greeting O		0.815	
Latency to approach O		-0.814	
Greeting S			0.754
Latency to approach S			-0.731
Eigenvalues	1.780	1.379	1.015
Explained variance (%)	25.672	24.502	19.388

*KMO* = 0.513; Bartlett: χ^2^ = 62.088, df = 15, p<0.000.

#### Memory test with distraction

In this subtest we measured only one variable “latency to find food”, so this was Z- transformed.

#### Detour

The PCA revealed that the three variables measuring the duration of time looking at E/O and latency to success in the 1^st^, 3^rd^ and 4^th^ trials grouped on one component, whereas staying close to the gate and latency to success in the 1^st^, 3^rd^ and 4^th^ trials grouped on another component (Table 9). Latency to success in the 1^st^, 3^rd^ and 4^th^ trials had cross loadings in both components. We labelled the first component as “*Help seeking*” since the variables were related to the dog looking for help to solve the detour while the second component was labelled as “*Perseverance*”, since the variables loading on this component revealed how persistent dogs’ were in remaining at the gate and how much they were focused on reaching the food. These two components explained 73.90% of the observed variation in the data.

**Table 9:** Components derived from PCA on the variables measured in the detour subtest.

Variable	Help seeking	Perseverance
Duration of looking at E/O in trial 1	0.893	
Duration of looking at E/O in trial 3	0.798	
Latency to success in trial 1	0.741	0.475
Duration of looking at E/O in trial 4	0.711	
Latency to success in trial 4	0.641	0.604
Latency to success in trial 3	0.619	0.605
Duration of being close to gate in trial 3		0.920
Duration of being close to gate in trial 4		0.806
Duration of being close to gate in trial 1		0.710
Eigenvalues	5.37	1.11
Explained variance (%)	38.80	35.12

*KMO* = 0.635; Bartlett: χ^2^ = 786.44, df = 36, p < 0.001.

#### Attention

A PCA on the measured variables for this subtest revealed that they grouped on one component explaining a variance of 55.69%. We labelled this component ‘*Attentiveness’*, as the variables that strongly loaded on it all measured dogs’ attentiveness towards the stimuli and not O (Table 10).

**Table 10:** Component derived from PCA on the variables measured in the attention subtest.

Variable	Attentiveness
Total duration of looking at human	0.772
Total duration of looking at O in human condition	-0.766
Total duration of looking at toy	0.733
Total duration of looking at O in toy condition	-0.713
Eigenvalue	2.23
Explained variance (%)	55.69

*KMO* = 0.618; Bartlett: χ^2^ = 93.36, df = 6, p < 0.001.

#### Novel action

The variable “latency to pull out board in the first trial” was Z- transformed. This Z- transformed variable was multiplied by -1 to denote higher problem solving.

#### Manipulative persistency

A PCA on the measured variables for this subtest revealed that they grouped on one component explaining 55.69% of the variance. We labelled this component ‘*Motivation & persistency*’, as the variables measured dogs’ motivation to manipulate the toy and persistency in step 2 during the unsolvable trial (Table 11).

**Table 11:** Components derived from PCA on the variables measured in the manipulative persistency subtest.

Variables	Motivation & persistency
Percentage time of manipulating toy in step1	0.859
Percentage time of manipulating toy in step2	0.859
Eigenvalue	1.476
Explained variance (%)	73.777

*KMO* = 0.500; Bartlett: χ^2^ = 22.95, df = 1, p < 0.001.

#### Clicker training for eye contact

A PCA on the measured variables for this subtest revealed that they grouped on one component explaining 56.88% of the variance. We labelled this component ‘*Attention & trainability’*, as the variables measured selective attention and trainability (through the performance of dogs in the last three trials compared to first three trials) (Table 12). This component was multiplied by -1 to denote higher *Attention and trainability* (i.e. a shorter latency to eye contact and to find food).

**Table 12:** Component derived from PCA on the variables measured in the clicker training for eye contact subtest.

Variable	Attention & trainability
Latency to find food average of last 3 trials	0.849
Latency to eye contact average of first 3 trials	0.809
Latency to find food average of first 3 trials	0.733
Latency to eye contact average of last 3 trials	0.601
Eigenvalue	2.275
Explained variance (%)	56.88

*KMO* = 0.701; Bartlett: χ^2^ = 87.35, df = 6, p < 0.001.

### Factors defining underlying behaviour and cognition

The exploratory factor analysis (Kaiser-Meyer-Olkin Criterion = 0.504; Bartlett’s Test of Sphericity = 158.90, p< 0.001) resulted in 6 factors (Eigenvalue > 1), which account for 43.62% of the total variance in the data and included a total of 12 components (out of 13) plus two Z-transformed variables. The PCA component from the food choice subtest had a low commonality compared to the others (0.180), and therefore we excluded this component from the final EFA. Table 13 shows the factor loadings of the subtest components and variables in the final EFA. The factors were given labels to summarize the subtest components that they contained.

**Table 13:** Results of the exploratory factor analysis (EFA).

Subtest name	Subtest component name	Factors					
		Problem solving	Sociability	Trainability	Boldness	Activity-independence	Dependency
**Exploration**	*Activity/ Exploration*	-0.014	0.188	-0.146	0.047	**0.619**	0.037
**Picture viewing**	*Independence*	0.068	-0.200	0.114	0.107	**0.663**	-0.020
**Picture viewing**	*Dependency*	0.032	**0.401**	**-0.342**	0.266	0.182	**0.492**
**Separation**	*Resisting Separation*	-0.021	-0.075	0.018	-0.056	0.048	**0.419**
**Greeting & playing**	*Dependency*	-0.085	0.024	0.075	0.032	-0.065	**0.483**
**Greeting & playing**	*Playfulness*	0.133	**0.827**	0.247	-0.023	-0.016	-0.064
**Greeting & playing**	*Openness*	-0.097	-0.015	0.000	**0.735**	-0.014	-0.139
**Memory test with distraction**	*Searching behaviour (Memory)*	-0.014	-0.025	-0.077	**-0.409**	-0.196	-0.132
**Detour**	*Perseverance*	-0.036	-0.203	**-0.437**	0.055	0.062	0.074
**Detour**	*Help seeking*	**-0.456**	-0.104	-0.044	0.254	-0.094	0.137
**Attention**	*Attentiveness*	**0.478**	-0.169	-0.112	-0.093	0.015	-0.014
**Novel action**	*Problem solving*	**0.669**	0.149	0.181	0.030	-0.145	0.006
**Manipulative persistency**	*Motivation & persistency*	**0.511**	0.200	-0.046	0.266	0.174	-0.082
**Clicker training for eye contact**	*Attention & trainability*	-0.021	-0.017	**0.775**	0.220	0.083	0.306
**Eigen value**		**1.990**	**1.89**	**1.60**	**1.34**	**1.24**	**1.15**
**Explained variance (%)**		**8.48**	**7.64**	**7.61**	**7.08**	**6.90**	**5.84**
**Cronbach’s alpha**		**0.56**	**0.24**	**0.37**	**0.46**	**0.51**	**0.47**

Loadings > 0.32 are highlighted in bold.

The first factor included a shorter latency to pull out the board in the novel action subtest, higher manipulation and persistency in the manipulative persistency subtest, higher attentiveness in the attention subtest and low help seeking behaviour and shorter latency to success in the detour subtest. Considering that all these factors measure the dogs’ ability to solve different tasks, we labelled it as “Problem solving”. The second factor, labelled as “Sociability”, was composed of the “*Playfulness”* component from the greeting and playing subtest and the *“Dependency”* component from the picture viewing subtest. The third factor was composed of a shorter latency to establish eye contact with E and a shorter latency to find dropped food on the floor in the clicker training for eye contact subtest, spending less time at the gate while solving the detour in the detour subtest and showing less dependency towards O in the picture viewing test. Since these components measured how attentive dogs were and how quickly they performed the tasks, we labelled this factor as “Trainability”. The fourth factor, labelled as “Boldness”, was composed of a stranger greeting component from the greeting and playing subtest and a shorter latency to find hidden food in the memory test. Since both these components require an openness to interact with a novel person or environment, we considered this behaviour as a sign of boldness. The fifth factor included the *“Activity/Exploration”* component from the exploration subtest and the *“Independence”* component from the picture viewing subtest, and was therefore labelled as “Activity-independence”. Finally, the sixth factor labelled as “Dependency” was composed of the *“Dependency”* components from the picture viewing subtest and the greeting and playing subtest and resisting separation from the owner in the separation subtest. The internal consistency (Cronbach’s alpha) of the factors was low. However, low consistency was expected because each factor contained only a few components, which is known to produce low Cronbach’s alpha values [73]. The factor scores had lower variance, as it had already been reduced in the subtest level PCAs. Three cross loadings in the EFA indicated that some of the components were correlated despite the varimax rotation (Table 13).

### Statistical models on the factors to determine the effects of age, diet and lifelong training

**Fig 1:**
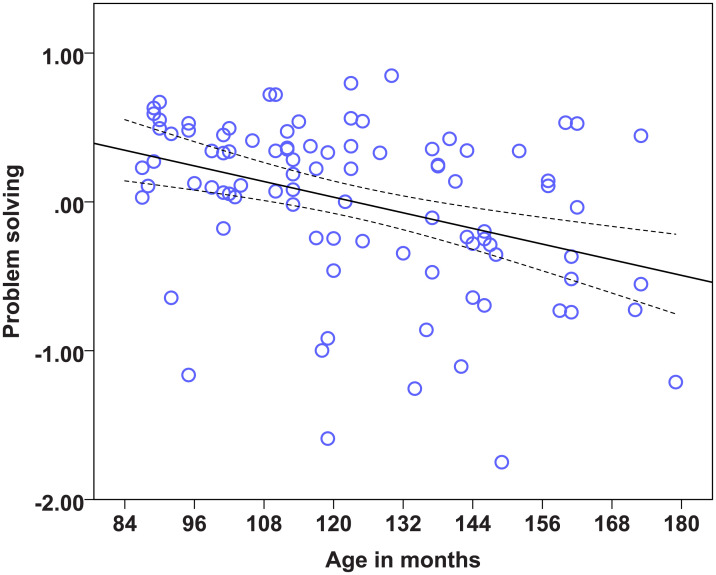
Scatter plots showing the relationship between age in months and problem solving [with 95% confidence intervals (dotted lines)]. A significant effect of age in months was present on the factor Problem solving (η^2^ = 0.15, p<0.0001).

**Fig 2:**
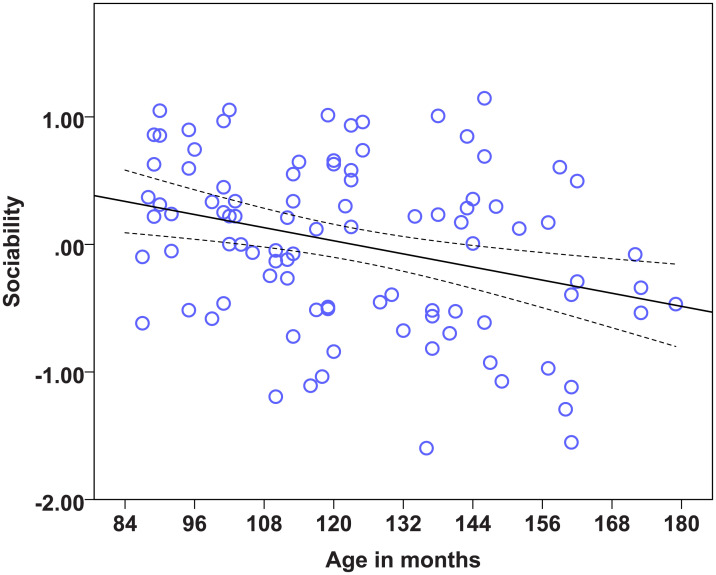
Scatter plots showing the relationship between age in months and sociability [with 95% confidence intervals (dotted lines)]. A significant effect of age in months was present on the factor Sociability (η^2^ = 0.10, p = 0.002).

**Fig 3:**
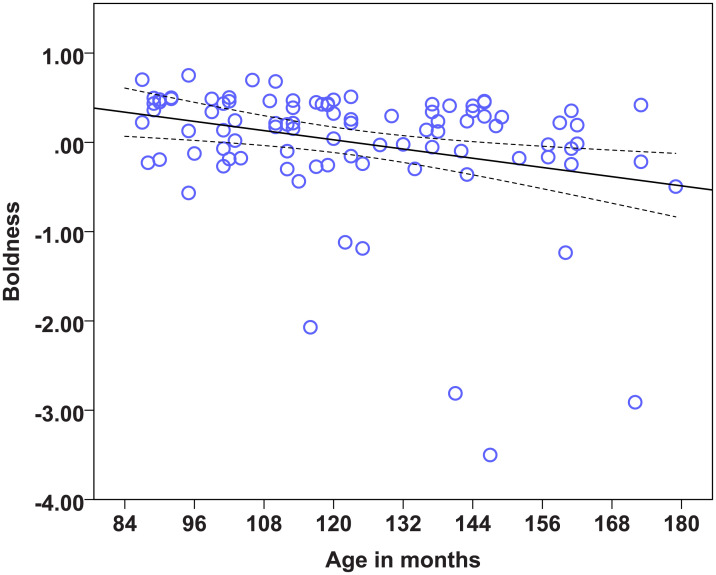
Scatter plots showing the relationship between age in months and boldness [with 95% confidence intervals (dotted lines)]. A significant effect of age in months was present on the factor Boldness (η^2^ = 0.10, p = 0.002).

**Fig 4:**
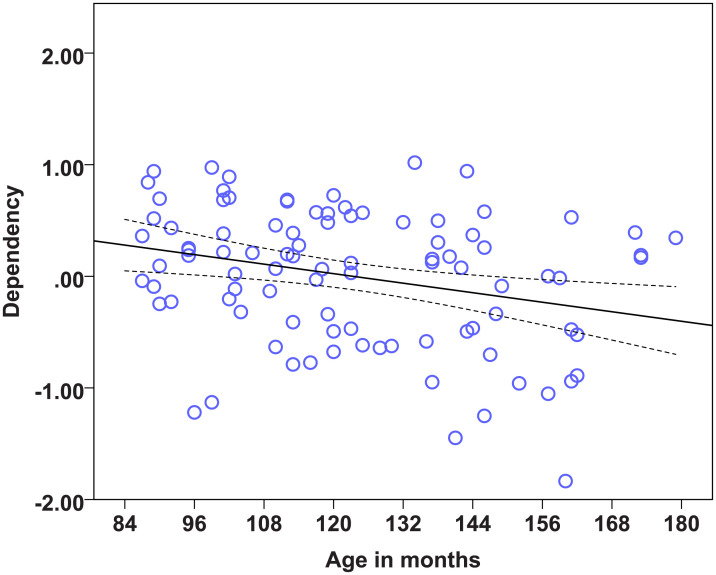
Scatter plots showing the relationship between age in months and dependency [with 95% confidence intervals (dotted lines)]. A significant effect of age in months was present on the factor Dependency (η^2^ = 0.07, p = 0.007).

**Fig 5:**
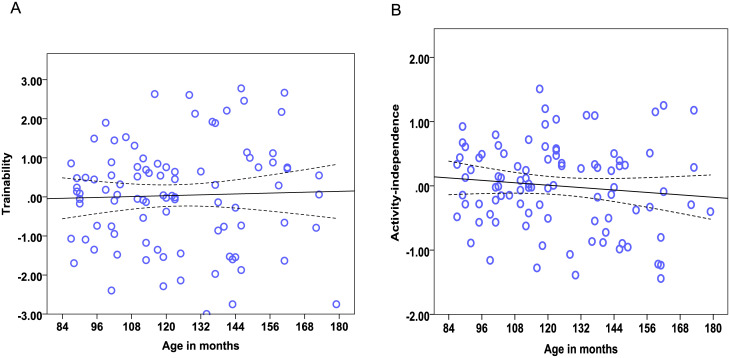
Scatter plots showing no change in Trainability (A) and Activity-independence (B) with increased age. The dotted lines show 95% confidence intervals.

We found strong age effects on the EFA factors measuring Problem solving, Sociability, Boldness, and Dependency (see Table 14). Regarding the dietary intervention, we found no effect of diet on any of the six factors. There was also no effect of lifelong training on any of the measured factors. Therefore, the most important predictor of the factor scores was found to be age. Dogs’ scores in the factor Problem solving were significantly influenced by age, as the Problem solving performance of dogs dropped with increasing age (Fig 1). Older dogs showed less sociable (Fig 2) but also less bold (Fig 3) and less dependent behaviour (Fig 4). Trainability and Activity-independence showed no change with increasing age (Fig 5A & 5B).

**Table 14:** Results of the full and reduced linear models on the six factors generated from the EFA.

Full model						Reduced model				
	Estimate	SE	t-value	p-value	Partial eta-squared	Estimate	SE	t-value	p-value	Partial eta-squared
**Factor1: Problem solving**										
Diet	1.425	1.044	1.366	0.176	0.010					
Age	-0.009	0.006	-1.472	0.145	0.164	-0.016	0.004	-4.099	**<0.0001**	0.150
Training score	-0.002	0.016	-0.121	0.904	0.000					
Age*diet	-0.014	0.008	-1.782	0.078	0.036					
Diet*training score	0.012	0.022	0.527	0.599	0.005					
**Factor2: Sociability**										
Diet	-0.155	0.707	-0.219	0.827	0.033					
Age	-0.008	0.004	-1.898	0.061	0.109	-0.009	0.003	-3.197	**0.002**	0.100
Training score	0.002	0.011	0.176	0.861	0.032					
Age*diet	-0.003	0.005	-0.497	0.621	0.003					
Diet*training score	0.022	0.015	1.489	0.140	0.026					
**Factor3: Trainability**										
Diet	1.203	1.546	0.778	0.438	0.003					
Age	0.009	0.009	0.995	0.322	0.001					
Training score	-0.023	0.024	-0.960	0.340	0.001					
Age*diet	-0.015	0.012	-1.277	0.205	0.018					
Diet*training score	0.043	0.033	1.298	0.198	0.022					
**Factor4: Boldness**										
Diet	-127.657	140.065	-0.911	0.365	0.001					
Age	-2.234	0.788	-2.836	**0.006**	0.103	-1.653	0.512	-3.226	**0.002**	0.100
Training score	-0.453	2.151	-0.210	0.834	0.004					
Age*diet	1.092	1.060	1.031	0.305	0.012					
Diet*training score	-1.282	2.992	-0.429	0.669	0.003					
**Factor5: Activity-independence**										
Diet	-1.043	0.774	-1.347	0.181	0.001					
Age	-0.007	0.004	-1.638	0.105	0.013					
Training score	0.001	0.012	0.089	0.929	0.013					
Age*diet	0.007	0.006	1.200	0.233	0.016					
Diet*training score	0.012	0.017	0.730	0.467	0.005					
**Factor6: Dependency**										
Diet	-0.758	0.906	-0.837	0.405	0.003					
Age	-0.013	0.005	-2.543	**0.013**	0.078	-0.009	0.003	-2.770	**0.007**	0.070
Training score	-0.005	0.014	-0.333	0.740	0.002					
Age*diet	-0.001	0.019	-0.063	0.950	0.000					
Diet*training score	0.007	0.007	1.007	0.317	0.012					

Significant predictors (p<0.05) are highlighted in bold.

## Discussion and conclusions

In this study, we examined how age, a diet enriched in antioxidants (Vitamin C, Vitamin E & Polyphenols), DHA, Phosphatidylserine and tryptophan, and lifelong training influence different behaviours and cognitive abilities of older pet dogs of various breeds. The exploratory factor analysis on the components from the subtests resulted in six factors, labelled as Problem solving, Trainability, Sociability, Boldness, Activity-independence and Dependency, each comprising the dogs’ behaviour in multiple situations, and capturing different behavioural and cognitive measures. Results revealed a significant effect of age on Problem solving, Sociability, Boldness, and Dependency. Surprisingly, we found no difference in any of the six factors between the groups with and without the enriched diet Lifelong training had no effect on any of the behavioural and cognitive measures.

The Problem solving factor we identified appears to be mostly influenced by cognitive abilities. It was comprised of a shorter latency to success in manipulative tasks, higher motivation and persistency to engage with a food toy, higher attentiveness and a lower tendency to seek help (gaze at E/O during the detour task). Such a component, “independent problem-solving/problem-orientation” has already been described by Bray et al. [74] and Turcsán et al. [35], as a cluster of behaviours that is comprised of problem solving ability, task focus and reduced help seeking behaviour. It is not surprising that the Problem solving factor also included motivation and persistency, being basic control processes that have been shown to strongly influence success in problem solving [75, 76]. Problem solving showed a linear decline with age in dogs older than 6 years. It should be noted that paying attention is considered as one of the most important components involved in problem solving. Based on previous studies, we have already been able to show that sustained attention [14] and selective attention [30] decline in dogs during aging. Hence, we consider it likely that the reduced problem solving ability in older dogs in our study group is due to this decline, as attentiveness was one component of the Problem solving factor. An alternative explanation could be that older dogs were less motivated and less persistent because of their underlying physical condition (motor/sensory deterioration) not evident in their full clinical and haematological evaluation, or they perceived the food reward as less valuable when compared to younger dogs, and thus invested less time and energy in fully solving the task (i.e. completely emptying the food toy or pulling out the board). It has been shown that higher quality rewards evoke greater incentive motivation than a greater quantity of a lower value reward in dogs [77]. Studies in humans show that older subjects appear to learn more slowly with low compared to high reward magnitudes, while younger subjects were not affected [78]. However, in our experience with different experiments that used various forms of reward in the CDL, dogs in general preferred sausage and cheese compared to other rewards. So, we are a little hesitant to emphasize the argument regarding the reward value for older dogs, although it might be a likely explanation. An age dependent decline in curiosity [19], or reduced interest in exploring objects and a lack of motivation related to food quality or reward magnitude, have also been suggested as possible explanations for the decline of problem solving in another cohort of dogs [79].

The factor Sociability was composed of variables such as playing with the stranger and owner, following the owner, and staying close to her/him and looking less at her/him. Items loading on this factor were associated with playfulness and owner attachment/dependency. Sociability as reported by Menchetti et al. [80] also included sociable and playfulness traits in dogs and cats. However, Sociability documented by Svartberg and Forkman [81] and Turcsán et al. [35] included dogs’ social behaviour towards strangers such as greeting and approach behaviour, and cooperation and handling. In our study, greeting and approach behaviours towards the stranger loaded on the factor Boldness. The dependency-related component of the Sociability factor had cross loadings with two other factors, Trainability and Dependency, while the playfulness component loaded strongly only on this factor. Sociability showed a linear decline with age in months. Hence, it clearly demonstrates that Sociability/*dependency* and *playfulness* were strongly affected by age, as in Menchetti et al. [80]. This result complements the widespread view that older dogs play less in comparison to younger dogs. Salvin et al. [82] showed a linear increase in the percentage of dogs that displayed deterioration in their time spent in playing in a cross-sectional sample of successfully aging dogs (≥ 8 years). Although dogs enrolled in our study were supposedly healthy as they were screened by a veterinarian at the time of inclusion and after one year and did not suffer from osteo-arthritis or cognitive dysfunction, the amount of playfulness showed a steady decline with age in this adult cohort. The documentation of a decline in play in aged dogs in Salvin et al. [82] was based on owners assessment with the help of questionnaires, which differs from our test to evaluate playfulness in dogs, however, the results complement each other.

Many old dogs suffer from age-related musculoskeletal degenerative conditions like osteo-arthritis and sarcopenia, and hence, due to physical discomfort, might be less inclined to play. One could also argue that a reduction in play in aged dogs may also be due to owners not engaging in play with older dogs anymore. In support of this, a decrease in off-lead activity and dog/owner interaction (including play) and training has been reported in dogs over 7 years by at least two studies [83, 84]. Despite this, we advocate that owners should actively invite their aged dogs to play (within their physical capabilities) to keep the dog-human bond more vibrant, and to maintain or increase activity levels which may help to prevent a decline in motor abilities.

The factor Boldness was composed of variables from two subtests, a stranger greeting component and a shorter latency to find hidden food, and as such it required willingness to interact with new stimuli, either a stranger or a new environment. Interestingly, the variable ‘latency to find food’ also included a memory component and hence the boldness factor subsumes both a behavioural and a cognitive measure. In previous studies, boldness in dogs has been characterized by exploration, willingness to play with humans, a low frequency and intensity of fearful behaviour directed towards humans and dogs, as well as non-social objects or events [81, 85, 86]. Specifically, when encountering new situations (new stimuli, new tasks), bold individuals tend to approach and explore novel stimuli quickly and more willingly [87, 88], which is also supported by our results. Previous studies have described boldness differently according to their research hypothesis, such as when examining the suitability of dogs’ in the working context [85, 86] or attempting to relate consistent individual differences in personality to individual differences in cognition (see review by Griffin et al. [88] and Sih and Giudice [88]). However, the factor Boldness that we identified in this study includes openness and memory, and showed levels decreasing in the older dogs. In line with our results, Starling et al. [25] also observed a decrease in boldness with increased age, however, their boldness factor was created from an owner questionnaire, and was comprised of more variables than our factor. One explanation for the reduction in boldness in older dogs could be that as dogs’ age and accumulate experiences, they engage less with their surroundings and show a reduction in excitement and/or curiosity, which may lead to behaviour that appears shier [24]. As such, they become less inclined to approach strangers or explore a new environment [25]. Age-related physical and cognitive degenerative processes could also lead to the expression of less bold behaviour in dogs [25], which cannot explain our results however, as in our sample we had no dogs that suffered from a physical or cognitive degenerative conditions.

The factor Dependency included variables such as approaching and greeting the owner, following the owner and staying in close proximity, resisting separation from the owner by staying close to the door (or looking at the door while sitting or standing passively) when the dog was left alone, and as such is thought to reflect the dog’s attachment to the owner. We consider dependency as an important basic control process in the sense that the dogs’ relationship with the owner might influence the dogs’ performance in different tasks. There was a decrease in dependency with increasing age of the dogs. Physiological changes during aging can affect the emotional and relational needs of old dogs which could thereby appear as altered dependency on their owner. As dogs’ age, owners engage in fewer shared activities (such as active training or play) with them [83, 84], which could also drive a decline in dependency.

Alternatively, older dogs may learn that the owner is very reliable, and as they age they worry less when left alone temporarily. As routine and predictability are two essential components of feeling safe, and older and therefore more experienced dogs show more relaxed behaviours when left alone in the room, although they are not necessarily less dependent. However, Mongillo et al. [83] argued that the passiveness observed in aged dogs during a separation episode in a behavioural test is potentially more active suppression of behavioural signs than a true relaxed reaction to social challenges, which was further supported by a significant increase in salivary cortisol concentrations in aged dogs after the test. Therefore, it is plausible to suggest that aging may signify a generalized increase in susceptibility to social and environmental stress, coupled with an increased suppression of behaviour, which resulted in the reduced Dependency scores found in older dogs. Moreover, dogs’ cognitive and behavioural changes during unsuccessful aging may affect the dog owner relationship in opposite ways, with older dogs showing either detachment to their owners [89] or alternatively becoming more clingy [90].

The factor Trainability included higher attentiveness and learning success, low perseverance and a lower latency to succeed in the detour task, and low dependency on the owner. Dogs’ trainability is more often evaluated based on their performance in obedience training, where the dogs must respond quickly and correctly to previously learned commands or directions given by their owner or handler [91]. More recently, trainability has been included in the personality assessment of dogs, usually through owner questionnaires [22–24] or measured in real life situations such as in a behavioural task [30]. Trainability basically involves a combination of willingness and focus to attend to the trainer (attentiveness/motivation/dependency), comprehension of what the training goal is (general cognitive ability) and the ability to remember the tasks being taught (memory) [92]. Thus, trainability seems to encompass both cognitive abilities and basic control processes. However, trainability measured through the questionnaires focused mainly on dogs’ playfulness [23] or behaviours other than playfulness [22–24]. Most interestingly, we found no change in Trainability with age. Studies conducted with questionnaires have documented mixed results [22–24]. Chopik and Weaver [24] reported that dogs around the age of 7–8 years show more responsiveness to training than younger dogs but are similar to dogs older than 8 years. Kubinyi et al. [23] however, reported that dogs below 3 years were more trainable than over 3. Wallis et al. [22] found that dogs older than 10 years tended to be less trainable than dogs aged 1–3 years but this difference became significant only above 12 years of age. Older dogs show declines in attentiveness [14, 30], memory [21] and executive functions [18], so a decline in responsiveness to training may be due to the combined influence of all these abilities. In contrast, our results suggest that older dogs remain trainable, and canine studies have shown that mental stimulation is an essential component in maintaining quality of life, and continued enrichment in the form of training, exercise, play and novel toys can help to maintain cognitive function [93]. Humans studies also stress the importance of maintaining/increasing mental activity and physical exercise in older humans as this can delay the onset of dementia [94, 95].

The Activity-independence factor was comprised of variables related to exploration, locomotion, moving independently and looking less at the owner, and as such it revealed how much the dog operated independently from the owner and explored its environment. Basically, activity reflects the general behavioural pattern of individuals, thus encompassing a behavioural aspect that can be affected by the severity of cognitive impairment [19], and musculo-skeletal problems [96]. Activity-independence was not affected by age in the current study. The dogs were all health checked and also categorized as successfully aging; so, as expected, we found no differences in their activity levels with advancing age. Similarly to our results, Rosado et al. [19] found that age did not affect locomotor activity, but the severity of cognitive impairment did, with severely impaired dogs showing higher locomotion. They describe this increased non-goal-directed locomotion as aimless walking, which is frequently observed in human dementia and canine CDS (Cognitive Dysfunction Syndrome: when severe) and possibly related with a dysfunction in the behavioural control mechanisms in the prefrontal cortical-basal ganglia circuitry. Moreover, a study in laboratory dogs also found no difference in the activity levels of younger and older dogs in an open field test similar to our results [97]. Nevertheless, studies that have used owners’ assessment of locomotor activity of pet dogs with the help of questionnaires documented a decrease in activity in older dogs compared to adults [89, 98]. In these questionnaires, owners were reporting general activity levels of dogs, which is not the same as in test batteries that examine activity over very short durations (1–2 minutes) in a novel environment.

Taken together, our test battery successfully demonstrated the individual behavioural variability in aged pet dogs that was likely influenced by several cognitive and basic control processes. Since the aim of our study was to measure the behavioural and cognitive variation relevant for the everyday life of dogs and owners, our study uniquely combined cognitive tasks with paradigms used to measure personality in dogs. Most former studies aimed to dissect either canine cognition [99–102] or personality [23, 81, 103, 104] using principle component analysis or exploratory factor analysis, similar to our methods. In this study, we combined tests of cognition and basic control processes. The findings that cognitive measures and behaviours typically linked to personality characteristics loaded together on most factors call attention to the fact that basic control processes (such as motivation, persistence and dependency) are likely to influence cognitive performance and behavioural variation linked to different personality traits [105].

Surprisingly, we found no effect of lifelong training on the obtained factors measuring cognitive abilities and basic control processes. In humans, the hypothesis that an active lifestyle that includes cognitive effort has long-term benefits for older adults’ cognition is consistent with the available data [42, 44]. In a similar note, we also found a positive effect of lifelong training experiences on different attention measures in pet dogs [14] but not in learning rate and cognitive flexibility [106]. Finally, the average lifelong training score for the dogs was quite low (11.67), which probably include only three training types, resulting in low variability which could also have affected the results. Future studies should examine training types individually to see if specific types influence cognition and basic control processes rather than a cumulative training score effect.

The lack of a diet effect in our study is in contrast to the majority of cross-sectional and longitudinal studies conducted in laboratory dogs, that have documented positive effects of antioxidant fortified food with additional supplementation of DHA, Phosphatidylserine and mitochondrial co-factors and a diet enriched with medium chain triglycerides on different cognitive measures [36, 48–52, 107, 108]. Furthermore, improvements in the symptoms of CDS in pet dogs have been reported with an enriched diet containing an antioxidant, DHA and mitochondrial co-factors [66] or a dietary supplementation with medium chain triglycerides, arginine, antioxidants, B vitamins and fish oil [67].

The lack of a diet effect in the current study may simply be due to the diet being ineffective, or to limitations of the study that prevented us from detecting the effect of the enriched diet. Before all, as demonstrated by a retrospective power analysis conducted to determine the sample size required to detect significant effects of enriched diet in each of the six EFA factors, we had inadequate power to detect a diet effect as the sample size was not adequate (see S5 Table in S1 File). Interestingly, a number of dietary intervention studies in laboratory dogs and a few studies in pet dogs have found a diet effect despite the majority of these studies did not address the issue of effect sizes (for the only exception see 68) and used sample sizes that were much lower than the sample used in our study [36, 48–52, 107, 108]. This discrepancy may be explained by the fact that other pet dog studies used older dogs and/or only dogs with at least two signs of CDS to show the effectiveness of enriched diet [66, 67]. As our subjects were relatively young (around 28% of the dogs were younger than 8 years when tested after one year of diet feeding) and we had no cognitively impaired dogs in our sample, the diet might have had a weaker effect in our study as compared to other studies. Furthermore, our sample was likely characterized with high inter-individual variation also in regard to housing conditions, amount of stimulation the dogs received living in different environments, and in regard to training and food treats that they got during their lifetime. Also the inclusion of several breeds including mixed breeds in the sample likely increased the variation in the data, thereby reducing the chance to find a diet effect with this sample size.

Nonetheless, it is worthwhile to mention the consideration that interventions are thought to be more effective in delaying deterioration when they are started early in the aging process. Given these preferences to provide younger dogs with potential treatments, we consider testing the effectiveness of interventions in healthy and normally aging dogs to be a valid and reasonable approach. We argue that, while dietary intervention studies performed on laboratory dogs may tell us whether or not a specific diet has a measurable effect in this subgroup of dogs, testing the effect of different diets on the general pet dog population will reveal its potential positive benefit to dogs living in the human society. Future studies should increase the sample size and test the dietary intervention not only in a sample of healthy and successfully aging pet dogs but also in a sample consisting of dogs older than 8 years and showing signs of cognitive dysfunction to disentangle whether or not the diet shows similar effect in both populations.

In conclusion, our test battery proved sensitive enough to evaluate individual differences across several cognitive measures and behavioural parameters and to detect age effects, even in such a sample of aging pet dogs that represents limited variation i.e successfully aging dogs. Importantly, we have also shown that the method of determining correlated individual variation across these tasks can be applied to detect age-related changes in pet dogs. So far, we do not have any objective measures to detect cognitive impairment in dogs except elaborate, time consuming and therefore impractical tests developed in laboratory beagles [18, 21, 48, 50, 65, 109], and so veterinarians rely on questionnaires and the owners’ assessment of their dog’s behaviour to screen cognitively impaired dogs. Therefore, the development of the MVCCB is a first step towards creating objective measures for identifying cognitively declining dogs using simpler, more efficient, and less time consuming experimental methods. This test battery should be further validated in dogs from veterinary clinics with owner-reported signs of cognitive impairment, and additionally in working dogs, service dogs and sports dogs, as it could be very useful for their care provider, and would allow the early detection of changes in behavioural and cognitive measures. It could be argued that the test battery will require substantial time and effort to code different behaviours from the tests; however, this test battery could be used as a reference to develop other simple tests that can be coded directly during testing, which will minimize the coding effort and be more realistic to test the dogs in the veterinary clinics. For example, the performance of dogs can be coded directly in the greeting and playing task, the food choice task and memory test. In a similar way, other tests like the detour task, manipulative persistency and novel action test can also be coded directly by modifying the test protocol.

So to summarize, using this test battery on a sample of pet dogs aged over 6 years of different breeds, we found a decline in Problem solving, Sociability, Boldness and Dependency with age. We did not detect any effect of a diet enriched with antioxidants (Vitamin C, Vitamin E & Polyphenols), DHA, Phosphatidylserine and tryptophan, or lifelong training on the different behavioural and cognitive measures in this study. Further studies are warranted to assess whether and how enriched diets and lifelong training may affect the aging of behavioural and cognitive skills in pet dogs.

## Supporting information

S1 File (DOC)
