# Supplementary material for: Behavioural and cognitive changes in aged pet dogs: No effects of an enriched diet and lifelong training
Source: PLoS One. 2020 Sep 16;15(9):e0238517. doi: 10.1371/journal.pone.0238517 (PMC7494100; doi:10.1371/journal.pone.0238517)
Supplement: S1 File — (DOC) [file pone.0238517.s001.doc]

**Supplementary materials**

***Behavioural and cognitive changes in aged pet dogs: no effect of enriched diet and lifelong training***

Durga Chapagain^1,2*^, Lisa J. Wallis^,3,4^, Friederike Range^2,1^, Nadja Affenzeller^5^, Jessica Serra^6^, Zsófia Virányi^1^

^1^Clever Dog Lab, Comparative Cognition, Messerli Research Institute, University of Veterinary Medicine, Vienna, Medical University of Vienna, University of Vienna, Vienna, Austria

^2^Domestication Lab, Konrad Lorenz Institute of Ethology, University of Veterinary Medicine, Vienna, Austria

^3^Department of Livestock and One Health, Institute of Infection, Veterinary and Ecological Sciences, University of Liverpool, UK

^4^Department of Ethology, Eötvös Loránd University, Budapest, Hungary

^5^Department/ Clinic for Companion Animals and Horses, University of Veterinary Medicine, Vienna, Austria

^6^ Royal Canin Research Centre, Aimargues, France

**Subjects:**

**Table S1: Individual characteristics of the 119 pet dogs that participated in the study.**

| **No.** | **Name** | **Breed** | **Sex** | **Trainingscore** | **Weight(Kg)** | **Diet** | **Age_**  **start_diet**  **(in months)** | **Age_**  **Final_testing**  **(in months)** | **Testing_status** |
| --- | --- | --- | --- | --- | --- | --- | --- | --- | --- |
| **1** | **Akira_staff** | American staff | F | 0 | 30 | test | 94 | 106 | **finished** |
| **2** | **Akita** | Mixed | F | 20 | 21 | test | 111 | 123 | **finished** |
| **3** | **Amadeus** | Cavalier King Charles Spaniel | M | 17 | 10 | test | 73 | 87 | **finished** |
| **4** | **Argon** | Swiss mountain dog | M | 1 | 30 | test | 80 | 95 | **finished** |
| **5** | **Avalon** | Beagle | F | 8 | 14.2 | test | 140 | 157 | **finished** |
| **6** | **Bastian** | Border collie | M | 8 | 25 | test | 108 | 123 | **finished** |
| **7** | **Bosco** | Mixed | M | 0 | 32.4 | test | 156 | 173 | **finished** |
| **8** | **Buddy** | Mixed | M | 8 | 15 | test | 148 | 161 | **finished** |
| **9** | **Darwin** | Border collie | M | 15 | 23.2 | test | 106 | 119 | **finished** |
| **10** | **Emiley** | Golden Retriever | F | 15 | 31 | test | 105 | 120 | **finished (only half)** |
| **11** | **Enigma** | Border collie | F | 18 | 20.3 | test | 137 | 152 | **finished** |
| **12** | **Fenja** | Mixed | F | 9 | 10 | test | 81 | 95 | **finished** |
| **13** | **Flamme** | French Pyrenean shepherd | M | 31 | 18 | test | 73 | 87 | **finished** |
| **14** | **Flora** | Golden Retriever | F | 12 | 37.3 | test | 103 | 120 | **finished** |
| **15** | **Flori** | Mixed | F | 28 | 17 | test | 123 | 137 | **finished** |
| **16** | **Foren** | Border collie | F | 12 | 16 | test | 97 | 110 | **finished** |
| **17** | **Frankie** | Golden Retriever | F | 0 | 32.1 | test | 86 | 102 | **finished** |
| **18** | **Fynn** | Poodle | M | 17 | 9.5 | test | 75 | 88 | **finished** |
| **19** | **Geena** | Border collie | F | 17 | 19 | test | 121 | 138 | **finished** |
| **20** | **Ginger** | Parson Russel Terrier | F | 19 | 10.1 | test | 106 | 123 | **finished** |
| **21** | **Guiness** | Border collie | F | 6 | 20 | test | 110 | 130 | **finished** |
| **22** | **Hybie** | Labrador Retriever | F | 0 | 28.3 | test | 78 | 90 | **finished** |
| **23** | **Ilvy** | Wolfspitz | F | 13 | 20.3 | test | 130 | 147 | **finished** |
| **24** | **Jack** | Mixed | M | 8 | 23.3 | test | 133 | 146 | **finished** |
| **25** | **Jacky** | Mixed | M | 11 | 13.5 | test | 98 | 112 | **finished** |
| **26** | **Kimba** | Mixed | F | 0 | 25 | test | 86 | 99 | **finished** |
| **27** | **Klara** | Mixed | F | 0 | 17 | test | 119 | 132 | **finished** |
| **28** | **Kora** | Mixed | F | 0 | 23 | test | 145 | 157 | **finished** |
| **29** | **Leah** | Border collie | F | 8 | 16 | test | 132 | 146 | **finished** |
| **30** | **Leila** | Mixed | F | 22 | 20 | test | 89 | 101 | **finished** |
| **31** | **Marlo** | Australian shepherd | M | 27 | 25 | test | 76 | 89 | **finished** |
| **32** | **Melissa** | German shepherd | F | 0 | 37.7 | test | 143 | 160 | **finished** |
| **33** | **Nash** | German shepherd | M | 23 | 34.2 | test | 108 | 125 | **finished** |
| **34** | **Nemo** | Labrador Retriever | M | 17 | 35.5 | test | 91 | 113 | **finished** |
| **35** | **Nero** | Miniature schnauzer | M | 0 | 9.5 | test | 122 | 134 | **finished** |
| **36** | **Picasso** | Mixed | M | 12 | 32.7 | test | 124 | 141 | **finished** |
| **37** | **Quinnie** | Malinois | F | 28 | 28.7 | test | 106 | 123 | **finished** |
| **38** | **Riga** | Entelbucher Mountain Dog | F | 4 | 18 | test | 89 | 101 | **finished** |
| **39** | **Scully** | Mixed | F | 8 | 28.7 | test | 96 | 113 | **finished** |
| **40** | **Shadow** | Mixed | M | 10 | 15.9 | test | 121 | 138 | **finished** |
| **41** | **Shane** | Belgian shepherd | F | 18 | 22 | test | 92 | 109 | **finished** |
| **42** | **Taiko** | American Akita | M | 12 | 33.7 | test | 80 | 95 | **finished** |
| **43** | **Tilly** | Parson Russel Terrier | F | 16 | 7 | test | 130 | 143 | **finished** |
| **44** | **Trisha** | Border collie | F | 2 | 25 | test | 146 | 162 | **finished** |
| **45** | **Wichtel** | Pyrenean shepherd | M | 14 | 11 | test | 129 | 142 | **finished** |
|  | **Peggy** | Australian shepherd | F | 15 | 22 | test | 109 |  | **dropped from diet** |
|  | **Sally** | Mixed | F | 5 | 19.5 | test | 100 |  | **dropped from diet** |
|  | **Apanatschi** | Australian shepherd | F | 9 | 21 | test | 97 |  | **dropped from diet** |
|  | **Charisma** | Weimaraner | F | 21 | 29 | test | 89 |  | **dropped from diet** |
|  | **Hotshot** | Mixed | M | 23 | 34 | test | 161 | 175 | **data discarded (fed herbal supplements)** |
|  | **Mozart** | Golden Retriever | M | 13 | 31.1 | test | 103 | 120 | **data discarded (fed fish oil)** |
|  | **Anjo** | Mixed | M | 18 | 10.2 | test | 84 |  | **dropped from study** |
|  | **Apryl** | Border collie | F | 14 | 18 | test | 74 |  | **dropped from study** |
|  | **Enia** | Dobermann | F | 8 | 32 | test | 122 |  | **dropped from study** |
|  | **Fleck** | Mixed | M | 5 | 32.6 | test | 84 |  | **dropped from study** |
|  | **Neo** | Mudi | M | 6 | 16 | test | 83 |  | **dropped from study** |
|  | **Roger** | Mixed | M | 12 | 18 | test | 168 |  | **died** |
|  | **Toby_corgi** | Mixed | M | 10 | 16 | test | 120 |  | **died** |
|  | **Geni** | Boxer | F | 7 | 20 | test | 98 |  | **died** |
|  | **Lord** | Golden Retriever | M | 7 | 31.5 | test | 164 |  | **died** |
| **1** | **Akira_mix** | Mixed | F | 21 | 23 | control | 106 | 119 | **finished** |
| **2** | **Ares** | Mixed | M | 12 | 27.3 | control | 144 | 162 | **finished** |
| **3** | **Arthur** | Mixed | M | 10 | 20 | control | 162 | 173 | **finished** |
| **4** | **Ayko** | Australian cattle dog | M | 12 | 24.9 | control | 108 | 125 | **finished** |
| **5** | **Cayenne** | Malinois | F | 14 | 28 | control | 145 | 159 | **finished** |
| **6** | **Chelsy** | Mixed | F | 0 | 42 | control | 98 | 117 | **finished** |
| **7** | **Chil** | Australian shepherd | F | 27 | 16 | control | 107 | 122 | **finished** |
| **8** | **Chilli** | Dutch shepherd | F | 34 | 24 | control | 135 | 148 | **finished** |
| **9** | **Cookie** | Mixed | F | 4 | 36 | control | 98 | 110 | **finished** |
| **10** | **Elina** | Mixed | F | 3 | 18 | control | 77 | 90 | **finished** |
| **11** | **Ella** | Munsterlander | F | 4 | 25 | control | 101 | 113 | **finished** |
| **12** | **Elvin** | Mixed | M | 0 | 9 | control | 77 | 89 | **finished** |
| **13** | **Fipsi** | Mixed | M | 10 | 10.7 | control | 144 | 161 | **finished** |
| **14** | **Ginger4** | Mixed | F | 10 | 9.1 | control | 120 | 137 | **finished** |
| **15** | **Gismo** | Border collie | M | 23 | 23.7 | control | 78 | 90 | **finished** |
| **16** | **Gwendolyn** | Swiss shepherd | F | 12 | 34 | control | 99 | 112 | **finished** |
| **17** | **Habibi** | Mixed | F | 0 | 16 | control | 127 | 140 | **finished** |
| **18** | **Havanna** | Beagle | F | 8 | 10 | control | 95 | 112 | **finished** |
| **19** | **Isla** | Podenco | F | 6 | 19 | control | 124 | 137 | **finished** |
| **20** | **Jersey** | Vizla | F | 10 | 22 | control | 85 | 99 | **finished** |
| **21** | **Jock** | Border collie | M | 12 | 22 | control | 89 | 103 | **finished** |
| **22** | **Leopold** | Mixed | M | 17 | 9 | control | 79 | 92 | **finished** |
| **23** | **Lestat** | Mixed | M | 4 | 32 | control | 129 | 143 | **finished** |
| **24** | **Luke** | Border collie | M | 27 | 24.3 | control | 102 | 114 | **finished** |
| **25** | **Mago** | Golden Retriever | M | 20 | 25.5 | control | 110 | 128 | **finished** |
| **26** | **Missface** | Mixed | F | 10 | 22 | control | 85 | 101 | **finished** |
| **27** | **Nanouk** | Australian shepherd | M | 15 | 21.1 | control | 77 | 92 | **finished** |
| **28** | **Nessie** | Mixed | F | 22 | 8.8 | control | 155 | 172 | **finished (only 9 test)** |
| **29** | **Nora** | Mixed | F | 18 | 20 | control | 103 | 117 | **finished** |
| **30** | **Nova** | Mixed | F | 16 | 22.4 | control | 86 | 104 | **finished** |
| **31** | **Patrash** | Mixed | M | 0 | 15 | control | 88 | 101 | **finished** |
| **32** | **Joy_GR** | Golden Retriever | F | 4 | 30 | control | 136 | 149 | **finished** |
| **33** | **Poris** | Mixed | M | 4 | 9.5 | control | 149 | 161 | **finished** |
| **34** | **Portos** | Mixed | M | 0 | 10 | control | 124 | 136 | **finished** |
| **35** | **Queenie** | Beagle | F | 4 | 13.1 | control | 103 | 120 | **finished** |
| **36** | **Rocky_lab** | Labrador Retriever | M | 12 | 29 | control | 84 | 96 | **finished** |
| **37** | **Rocky_mix** | Mixed | M | 2 | 17 | control | 89 | 102 | **finished** |
| **38** | **Ronja** | Parson Russel Terrier | F | 0 | 8 | control | 101 | 116 | **finished** |
| **39** | **Sammy** | Golden Retriever | M | 25 | 30 | control | 166 | 179 | **finished** |
| **40** | **Sayzi** | Mixed | M | 0 | 17 | control | 107 | 119 | **finished** |
| **41** | **Shari** | Golden Retriever | F | 15 | 32 | control | 88 | 103 | **finished (only half test)** |
| **42** | **Sidney** | Border collie | M | 19 | 23 | control | 96 | 113 | **finished** |
| **43** | **Sokrates** | Mixed | M | 22 | 31.4 | control | 93 | 110 | **finished** |
| **44** | **Sue** | Border collie | F | 13 | 19 | control | 76 | 89 | **finished** |
| **45** | **Sunny** | Border collie | M | 15 | 20 | control | 130 | 144 | **finished** |
| **46** | **Tika** | Mixed | F | 18 | 28.8 | control | 84 | 102 | **finished** |
| **47** | **Timi** | Vizla | F | 16 | 20 | control | 106 | 118 | **finished** |
| **48** | **Toby_lab** | Labrador Retriever | M | 12 | 27 | control | 132 | 144 | **finished** |
| **49** | **Todor** | Mixed | M | 10 | 15 | control | 132 | 146 | **finished** |
|  | **Aron** | Pitbull | M | 1 | 15 | control | 81 | 93 | **dropped from diet** |
|  | **Murphy** | Australian shepherd | M | 13 | 23 | control | 118 | 134 | **dropped from diet** |
|  | **Samara** | Mixed | F | 10 | 25 | control | 131 |  | **dropped from diet** |
|  | **Irmi** | Labrador Retriever | F | 11 | 31.2 | control | 107 | 127 | **data discarded (fed herbal supplements)** |
|  | **Kiwi** | Mudi | F | 17 | 16 | control | 81 | 95 | **data discarded (fed fish oil)** |
|  | **Hagar** | Australian shepherd | M | 30 | 22.4 | control | 123 | 140 | **data discarded (fed fish oil)** |
|  | **Karina** | Mudi | F | 8 | 12 | control | 107 |  | **dropped from study** |
|  | **Fibi** | Mixed | F | 10 | 20 | control | 74 |  | **dropped from study** |
|  | **Joy_GS** | German shepherd | F | 14 | 33 | control | 124 |  | **died** |
|  | **Pheobe** | Golden Retriever | F | 9 | 31 | control | 132 |  | **died** |

**Table S2. Distribution of the 119 pet dogs from 30 different breeds that were included in the Test and Control diet groups.**

| **No.** | **Breed types** | **Test diet** | **Control diet** | **No.** | **Breed types** | **Test diet** | **Control diet** |
| --- | --- | --- | --- | --- | --- | --- | --- |
| 1 | Mixed | 20 | 26 | 16 | Cavalier King Charles Spaniel | 1 | 0 |
| 2 | Border collie | 9 | 6 | 17 | Boxer | 1 | 0 |
| 3 | Australian shepherd | 3 | 4 | 18 | American staff | 1 | 0 |
| 4 | Golden retriever | 5 | 5 | 19 | American akita | 1 | 0 |
| 5 | German shepherd | 2 | 1 | 20 | Pitbull | 0 | 1 |
| 6 | White Swiss shepherd | 0 | 1 | 21 | Swiss mountain dog | 1 | 0 |
| 7 | Belgian shepherd | 1 | 0 | 22 | Entlebucher mountain dog | 1 | 0 |
| 8 | Dutch Shepherd | 0 | 1 | 23 | Miniature Schnauzer | 1 | 0 |
| 9 | Labrador | 2 | 3 | 24 | Berger des Pyrenees | 2 | 0 |
| 10 | Malinois | 1 | 1 | 25 | Poodle | 1 | 0 |
| 11 | Parson russel terrier | 2 | 1 | 26 | Doberman | 1 | 0 |
| 12 | Mudi | 1 | 2 | 27 | Podenco | 0 | 1 |
| 13 | Beagle | 1 | 2 | 28 | Münsterländer | 0 | 1 |
| 14 | Vizsla | 0 | 2 | 29 | Wolfspitz | 1 | 0 |
| 15 | Australian cattle dog | 0 | 1 | 30 | Weimeraner | 1 | 0 |

**Table S3.** **Descriptive statistics of the subjects (N=94) that completed the study, including age, weight and training score information.**

| **Diet** | **Age in months (Mean ± SD)** | **Weight in kg**  **(Mean ± SD)** | **Training score (Mean ± SD)** |
| --- | --- | --- | --- |
| **Test (n=45)** | 110.08±23.46 | 22.09±8.34 | 11.68±8.46 |
| **Control (n=49)** | 110.10±24.89 | 21.21±8.01 | 11.67±8.35 |

**Statistical analyses for MVCCB2 data**

Distribution of all 42 variables across the 11 subtests was plotted using histogram. Normality was assessed using Shapiro-Wilk test. We calculated mean and standard deviation of all the variables in each diet groups. Since most of the variables did not follow a normal distribution, we used non-parametric tests (Mann-Whitney U) to detect the differences between the test and the control diet groups (Table S4). We used cumulative link models for nominal variables in greeting and playing task.

We also carried out post-hoc power calculation for the generated 6 factors of EFA (Table S5). Because we were interested in whether an effect of diet was present and its effect size, we reduced the full model to a point where the effect of diet or a diet interaction was significant or as close to significant as possible. We then used G*Power (Erdfelder and Lang et al., 2007) to measure the effect size and power of the diet variable for each of the six different factors. Test family: F Test, Statistical test: Linear multiple regression: Fixed model, R2 increase, Type of power analysis: Post hoc: compute achieved power. Using the estimated effect size information for diet, we also determined required sample size to attain 0.80 power (A-priori: compute required sample size).

**Results:**

**Table S4. Comparison of all the raw variables between the Test and C**ontrol diet groups.

| **Tests** | **Diet** | |  |
| --- | --- | --- | --- |
|  | **Test (mean ± SD)** | **Control (mean ± SD)** | **Statistics** |
| **Exploration** |  |  |  |
| Percentage time of locomotion | 69.08±20.75 | 70.42±17.53 | Z=-0.004, p=0.99 |
| Percentage time of exploration | 35.96±19.46 | 30.43±15.49 | Z=-1.53, p=0.12 |
| Percentage time of looking at O | 5.10±4.89 | 8.55±9.32 | Z=-1.57, p=0.11 |
| Percentage time of within 1m of O | 16.13±15.83 | 17.41±17.58 | Z=-0.28, p=0.77 |
| Percentage time of being inactive | 20.12±23.33 | 21.02±19.64 | Z=-0.50, p=0.61 |
|  |  |  |  |
| **Picture viewing** |  |  |  |
| Duration of time within 1m of O | 26.76±16.34 | 27.70±14.14 | Z=-0.05, p=0.95 |
| Duration of following O | 22.28±11.73 | 22.83±10.71 | Z=-0.41, p=0.67 |
| Duration of looking at O | 13.26±12.85 | 12.46±13.22 | Z=-0.49, p=0.62 |
| Duration of moving independently | 7.44±6.48 | 8.53±9.75 | Z=-0.32, p=0.75 |
|  |  |  |  |
| **Food choice** |  |  |  |
| Number of choices of baited plate in step1 | 4.17±1.24 | 4.48±1.13 | Z=-1.13, p=0.25 |
| Number of choices of baited plate in step2 | 3.97±1.55 | 4.22±1.55 | Z=-0.75, p=0.45 |
|  |  |  |  |
| **Separation** |  |  |  |
| Duration of looking at door | 40.02±37.13 | 33.77±36.22 | Z=-0.31, p=0.75 |
| Duration of positioned at door | 57.65±42.84 | 60.90±46.36 | Z=-0.96, p=0.33 |
| Duration of locomotion | 19.81±13.87 | 22.91±19.77 | Z=-0.06, p=0.94 |
| Duration of exploration | 2.70±3.80 | 4.44±7.53 | Z=-0.11, p=0.91 |
|  |  |  |  |
| **Greeting and playing** |  |  |  |
| Latency to approach stranger | 4.02±6.00 | 2.85±5.40 | Z=-1.11, 0.26 |
| Greeting stranger |  |  |  |
| score 0 | 24.44% | 18.37% |  |
| score 1 | 22.22% | 26.53% | Z=0.10, p=0.91 |
| score 2 | 44.44% | 55.10% |  |
| score 3 | 8.89% | 0% |  |
| Latency to approach owner | 3.06±4.63 | 2.32±4.90 | Z=-1.72, p=0.08 |
| Greeting owner |  |  |  |
| score 0 | 4.44% | 2.04% |  |
| score 1 | 6.67% | 4.08% | Z=-0.47, p=0.63 |
| score 2 | 62.22% | 75.51% |  |
| score 3 | 26.67% | 18.37% |  |
| Playing with stranger |  |  |  |
| score 0 | 8.89% | 32.65% |  |
| score 1 | 0% | 4.08% | Z=-1.52, p=0.12 |
| score 2 | 8.89% | 8.16% |  |
| score 3 | 82.22% | 55.10% |  |
| Playing with owner |  |  |  |
| score 0 | 46.67% | 65.31% |  |
| score 1 | 8.89% | 4.08% | Z=-3.29, p=0.0009 |
| score 2 | 4.44% | 4.08% |  |
| score 3 | 40.00% | 26.53% |  |
|  |  |  |  |
| **Memory test with distraction** |  |  |  |
| Latency to find food | 10.34±22.39 | 11.61±24.45 | Z=-0.43, p=0.66 |
|  |  |  |  |
| **Detour** |  |  |  |
| Duration of looking at E/O in trial 1 | 8.57±13.93 | 8.51±14.52 | Z=-0.28, p=0.77 |
| Duration of looking at E/O in trial 3 | 6.48±13.16 | 10.86±17.91 | Z=-1.26, p=0.20 |
| Duration of looking at E/O in trial 4 | 5.61±13.34 | 9.81±20.77 | Z=-0.69, p=0.49 |
| Latency to success in trial 1 | 51.22±46.85 | 53.64±47.59 | Z=-0.28, p=0.77 |
| Latency to success in trial 3 | 43.97±43.54 | 55.91±49.89 | Z=-0.60, p=0.54 |
| Latency to success in trial 4 | 26.45±37.73 | 36.99±45.85 | Z=-0.26, p=0.79 |
| Duration of being close to gate in trial 1 | 21.28±30.18 | 25.86±29.74 | Z=-1.69, p=0.09 |
| Duration of being close to gate in trial 3 | 23.01±33.39 | 25.89±33.84 | Z=-0.35, p=0.97 |
| Duration of being close to gate in trial 4 | 11.14±19.31 | 10.59±18.96 | Z=-0.14, p=0.88 |
|  |  |  |  |
| **Attention** |  |  |  |
| Total duration of looking at human | 36.17±16.17 | 38.40±15.86 | Z=-0.707, p=0.48 |
| Total duration of looking at toy | 26.49±17.23 | 24.14±15.24 | Z=-0.52, p=0.60 |
| Total duration of looking at O in toy condition | 10.42±12.64 | 11.05±11.71 | Z=-0.75, p=0.45 |
| Total duration of looking at O in human condition | 5.63±9.05 | 5.56±8.65 | Z=-0.29, p=0.76 |
|  |  |  |  |
| **Novel action** |  |  |  |
| Latency to pull out board | 62.35±78.64 | 74.09±101.22 | Z=-0.38, p=0.700 |
|  |  |  |  |
| **Manipulative persistancy** |  |  |  |
| Percentage time of manipulating toy in step1 | 79.03±11.72 | 76.70±12.72 | Z=-0.92, p=0.35 |
| Percentage time of manipulating toy in step2 | 67.39±23.42 | 64.48±20.62 | Z=-0.83, p=0.40 |
|  |  |  |  |
| **Clicker training for eye contact** |  |  |  |
| Latency to eye contact average of first 3 trials | 8.34±7.77 | 7.83±6.83 | Z=-0.54,p=0.58 |
| Latency to eye contact average of last 3 trials | 5.24±4.32 | 5.68±5.28 | Z=-0.25, p=0.80 |
| Latency to find food average of first 3 trials | 2.16±3.41 | 1.63±0.92 | Z=-1.34, p=0.17 |
| Latency to find food average of last 3 trials | 1.90±1.27 | 1.77±1.80 | Z=-1.81, p=0.06 |

**Table S5.** Table showing post-hoc power calculation for the effect of diet. A priori sample size is also determined.

| **Response variable** | **Sample Size** | **R squared** | **Model code** | **Sign effects in model** | **Diet Predictor** | **Diet predictor P value** | **Partial eta** | **Power** | **A-priori sample size** |
| --- | --- | --- | --- | --- | --- | --- | --- | --- | --- |
| Problem solving | 92 | 0.196 | age+diet+training+age:diet | Age | Age*diet | 0.07 | 0.036 | 0.527 | 178 |
| Sociability | 94 | 0.128 | age + diet | Age | Diet | 0.09 | 0.031 | 0.343 | 223 |
| Trainability | 94 | 0.026 | age + diet + training + diet:training | None | Training*diet | 0.165 | 0.022 | 0.231 | 335 |
| Boldness | 94 | 0.117 | age+diet+training+age:diet | Age | Age*diet | 0.286 | 0.013 | 0.163 | 536 |
| Activity-independence | 94 | 0.053 | age+diet+training+age:diet | None | Age*diet | 0.337 | 0.01 | 0.129 | 746 |
| Dependency | 94 | 0.092 | age+diet+training+age:diet | Age | Age*diet | 0.31 | 0.012 | 0.151 | 596 |

**Methods of MVCCB 1**

All the 11 subtests included in MVCCB1 were performed in a room measuring 7.125 x 6 meters at the Clever Dog Lab. The same experimenter (hereafter “E”; main author DC) conducted all the subtests. In the testing room, two doors were located approximately two meters apart on the front wall. One door was designated as the owner (hereafter “O”) door, and she/he used this door exclusively to enter and exit the room, the other door was E’s door. The room contained only the equipment required for the actual test. Owners were instructed verbally to follow the experimenter’s instructions for each subtest and were additionally handed a written test protocol, which they read before each subtest.

1. **Exploration**

The room was enriched with few objects like umbrella, a cartoon box with some papers, an empty bottle, a bag with some magazines, a table with table cloth, a chair and a table with a closed box of sausage on top. The detail description of the test and variables measured were similar to exploration test of MVCCB 2.

1. **Picture Viewing**

The details of the test and variables measured were similar to picture viewing test of MVCCB 2.

1. **Food choice**

The details of the test and variables measured were similar to food choice task of MVCCB 2. The only difference in the methodology was that here we used white colour plates compared to yellowish green colour plates used in MVCCB 2.

1. **Separation**

The details of the test and variables measured were similar to separation test of MVCCB 2.

1. **Greeting and playing**

The details of the test and variables measured were similar to greeting and playing task of MVCCB 2. In this test, E played the role of stranger.

1. **Memory test with distraction**

The details of the test and variables measured were similar to the food search task of MVCCB 2. However, more than 30 dogs were tested after only 2 minutes of waiting time outside.

1. **Attention**

In this test, the type and presentation of the stimuli were different from MVCCB 2. Instead of toy train and moving human in circles, flying toy and human pretending to paint an invisible wall were presented to the dog. In the toy condition, after the O and the dog were in position, E pulled a fishing line from outside to lift the toy up from the floor and then moved it up and down in the centre of the room. By watching the dog on the outside screen, E made sure that she started to move the toy when the dog was looking away from it. The toy was moved up and down in front of the dog for two minutes. After two minutes, E fixed the toy to the ceiling and signalled the O to come outside. In human condition, after the O and the dog were in position and the dog looked away from the door, E entered the testing room, closed the door, walked to a blue cross on the floor marked at a distance of 3m from the dog and started to move up and down vertically, pretending to paint an invisible wall for 2 min. While moving, she had her back to the dog so that the dog had no chance to establish eye contact with her. After two minutes, E went outside and then signalled the owner to leave the room. In this test, we measured only two variables: duration of looking at toy and duration of looking at human. For detail description of test, plz see Chapagain et al. (2017).

1. **Selective attention**

This test included owner and experimenter walking protocol which was adapted from Mongillo et al. (2010)**.** In this test, we measured selective attention of dogs towards the O and E. The O went inside the room with dog, took off the leash and did some tricks or commands dog knows for given 20 pieces of sausage. After two minutes when the E knocked on the door, the O tied the dog on red leash attached to the wall and came outside the room. E and O then waited for 30sec and started to move from one door to the other which was 1 m apart. O walked into the room from one door and E walked at the same time from another door. Both of them kept on walking slowly from one door to the other door while avoiding eye contact with the dog. Both of them walked according to the following sequence:

(1) Owner and E entered the room, walked to the opposite door, then turned back and took exit from the first door

(2) Owner and E entered the room, walked to the opposite door and left the room

(3) Owner and E entered the room, walked to the opposite door and left the room

(4) Owner and E entered the room, walked to the opposite door and left the room

(5) Owner and E entered the room, walked to the opposite door, then turned back and took exit from the first door.

After finishing the sequence, both doors were closed and the dog was left alone for 30 sec after which the test was finished.

**Variables measured:**

**Looking at owner** – Percentage time looking at the owner (when the owner is visible and also if the owner is not visible and dog is looking at the door from where the owner last went out).

**Looking at experimenter**- Percentage time looking at the experimenter (when the experimenter is visible and if the experimenter is not visible and dog is looking at the door from where the experimenter last went out).

1. **String pulling ( Novel action)**

**Test Apparatus**

The apparatus used for this experiment consisted of a fence covered with a black curtain which was positioned in one corner of the experimental room. The experimenter sat behind the fence, and pushed out one blue knotted string with sausage attached to the end positioned underneath the fence. The string was positioned so that the dog can either paw or mouth the string to pull the string out to get the sausage. The string was positioned either on the left or the right side, and the order of first appearance was counterbalanced among dogs.

**Procedure**

The owner walked to the center of the room, removed the leash, and sat down on the chair and let the dog free to explore the room. The experimenter was positioned inside the fence and was not visible to the dog or owner. After 1 minute, E pushed out a string from behind the fence, so that a small part of the string was visible to the dog. The sausage was not visible but the dogs could smell the sausage. The dog needed to use its paw or mouth to pull out the string to obtain the food. In the first trial, the food was placed close to the dog, so that he/she only needed to pull the string a few inches to obtain the reward. But from the 2^nd^ trial, sausage was placed further away from the string so that dog had to pull the string until the sausage was visible. When the dog successfully pulled the string out fully once on one side of the apparatus, the E swapped the string over to the other side of the apparatus (fence), and the dog had to pull the string out in the same way to get the reward. Each time the dog had finished pulling out the string, and had eaten the reward, the owner said “ok”, and the string was pulled in by the E. The trail was repeated for 6 times if the dog was successful in pulling string in first trial. In every trial, owner could encourage the dog after 30s of the appearance of string. The test criteria were set at a total of seven successful pull outs of the string, (one warm up trial, and three on the right and three on the left hand side). But if the dog was not successful to pull out the string in 5 minutes then the test was finished.

**Variables:**

We measured the latency to pull the rope in trial one and six subsequent trials; however we used only the measurement of the first trial due to the fact that dogs varied in the number of trials completed.

1. **Manipulative persistency**

The details of the tests and variables measured were similar to manipulative persistency task of MVCCB 2. The only difference was that we used a “Kong Wobbler” in this task and filled it with small pieces of dry food in step1 and bigger pieces of dry food in step2 in contrast to cheese used in MVCCB 2 manipulative persistency task.

1. **Clicker training for eye contact**

The details of the tests and variables measured were similar to clicker training for eye contact task of MVCCB 2.

**Results of MVCCB 1**

Variables included in MVCCB 1 were analysed only if diet effect was detected in any of the 6 factors of EFA. Since we did not detect any diet effect on the generated factor scores of EFA, we did not include the results of MVCCB 1 in the manuscript.

**References**

Chapagain, D., Virányi, Z., Wallis, L. J., Huber, L., Serra, J., and Range, F. (2017). Aging of Attentiveness in Border Collies and Other Pet Dog Breeds: The Protective Benefits of Lifelong Training . *Front. Aging Neurosci.*  9, 100. Available at: http://journal.frontiersin.org/article/10.3389/fnagi.2017.00100.

Faul, F., Erdfelder, E., Lang, A. et al. G*Power 3: A flexible statistical power analysis program for the social, behavioral, and biomedical sciences. Behavior Research Methods 39, 175–191 (2007). https://doi.org/10.3758/BF03193146

Mongillo, P., Bono, G., Regolin, L., and Marinelli, L. (2010). Selective attention to humans in companion dogs, Canis familiaris. *Anim. Behav.* 80, 1057–1063. doi:10.1016/j.anbehav.2010.09.014.
